# Forecast evaluation for data scientists: common pitfalls and best practices

**DOI:** 10.1007/s10618-022-00894-5

**Published:** 2022-12-02

**Authors:** Hansika Hewamalage, Klaus Ackermann, Christoph Bergmeir

**Affiliations:** 1 School of Computer Science & Engineering, University of New South Wales, Sydney, Australia; 2 SoDa Labs and Department of Econometrics & Business Statistics, Monash Business School, Monash University, Melbourne, Australia; 3 Department of Data Science and AI, Faculty of IT, Monash University, Melbourne, Australia

**Keywords:** Time series forecasting, Forecast evaluation

## Abstract

Recent trends in the Machine Learning (ML) and in particular Deep Learning (DL) domains have demonstrated that with the availability of massive amounts of time series, ML and DL techniques are competitive in time series forecasting. Nevertheless, the different forms of non-stationarities associated with time series challenge the capabilities of data-driven ML models. Furthermore, due to the domain of forecasting being fostered mainly by statisticians and econometricians over the years, the concepts related to forecast evaluation are not the mainstream knowledge among ML researchers. We demonstrate in our work that as a consequence, ML researchers oftentimes adopt flawed evaluation practices which results in spurious conclusions suggesting methods that are not competitive in reality to be seemingly competitive. Therefore, in this work we provide a tutorial-like compilation of the details associated with forecast evaluation. This way, we intend to impart the information associated with forecast evaluation to fit the context of ML, as means of bridging the knowledge gap between traditional methods of forecasting and adopting current state-of-the-art ML techniques.We elaborate the details of the different problematic characteristics of time series such as non-normality and non-stationarities and how they are associated with common pitfalls in forecast evaluation. Best practices in forecast evaluation are outlined with respect to the different steps such as data partitioning, error calculation, statistical testing, and others. Further guidelines are also provided along selecting valid and suitable error measures depending on the specific characteristics of the dataset at hand.

## Introduction

In the present era of Big Data, Machine Learning (ML) and Deep Learning (DL) based techniques are driving the automatic decision making in many domains such as Natural Language Processing (NLP) or Time Series Classification (TSC, Bagnall et al. 2016; Fawaz et al. 2019). Although fields such as NLP and Computer Vision have heavily been dominated by ML and DL based techniques for decades by now, this has hardly been the case for the field of forecasting, until very recently. Forecasting was traditionally the field of statisticians and econometricians. However, with massive scales of data being collected nowadays, ML and DL has now emerged as the state of the art for many forecasting tasks. Furthermore, with many companies hiring data scientists, often these data scientists are tasked with forecasting. Therefore, now in many situations practitioners tasked with forecasting have a good background in ML and data science, but are less aware of the decades of research in the forecasting space. This involves many aspects of the process of forecasting, from the point of data pre-processing, building models to final forecast evaluation. Due to the self-supervised and sequential nature of forecasting tasks, it is often associated with many pitfalls that usual ML practitioners are not aware of. The usage of bad evaluation practices worsens this problem since they are not clearly distinguishing the truly competitive methods from the inferior ones by avoiding spurious results. Evaluating the performance of models is key to the development of concepts and practices in any domain. Hence, in this particular work, we focus on the evaluation of point forecasts as a key step in the overall process of forecasting.

The general process of forecast evaluation involves employing a number of models having different characteristics, training them on a training dataset and then applying them on a validation set afterwards. Then, model selection may be performed by evaluating on the validation set to select the best models. Otherwise, ensemble models may be developed instead, by combining the forecasts from all the different models, and usually a final evaluation is then performed on a test set (Godahewa et al. 2021). In research areas such as classification and regression, there are well-established standard practices for evaluation. Data partitioning is performed by using a standard k-fold Cross-Validation (CV) to tune the model hyperparameters based on the error on a validation set, the model with the best hyperparameter combination is tested on the testing set, standard error measures such as squared errors, absolute errors or precision, recall, or area under the curve are computed and finally the best models are selected. These best methods may continue to deliver reasonable predictions for a certain problem task, i.e., they generalize well, under the assumption that there are no changes of the distribution of the underlying data, which otherwise would need to be addressed as concept drift (Webb et al. 2016; Ghomeshi et al. 2019; Ikonomovska et al. 2010) or non-stationarity.

In contrast, evaluating forecasting models can be a surprisingly complicated task, already for point forecasting. Data partitioning has many different options in the context of forecasting, including fixed origin, rolling origin evaluation and other CV setups as well as controversial arguments associated with them. Due to the inherent dependency, non-stationarity and non-normality of time series, these choices are complex. Also, most error measures are susceptible to break down under certain of these conditions. Other considerations are whether to summarize errors across all available time series or consider different steps of the forecast horizon separately etc. As a consequence, we regularly come across papers in top Artificial Intelligence (AI)/ML conferences and journals (even winning best paper awards) that use inadequate and misleading benchmark methods for comparison (e.g., non-seasonal models for long-term forecasting on seasonal series), others that use mean absolute percentage error (MAPE) for evaluation with series, e.g., with values in the $[-1,\ 1]$ interval because the authors think the MAPE is a somewhat generic “time series error measure”, even though MAPE is clearly inadequate in such settings. Other works make statements along the lines of Auto-Regressive Integrated Moving Average (ARIMA) being able to tackle non-stationarity whereas ML models can’t, neglecting that the only thing ARIMA does is a differencing of the series as a pre-processing step to address non-stationarity. A step that can easily be done as preprocessing for any ML method as well. In other works, we see methods compared using Mean Absolute Error (MAE) as the error measure, and only the proposed method by those authors is trained with L1 loss, all other competitors with L2 loss, which leads to unfair comparisons as the L1 loss optimizes towards MAE, whereas the L2 loss optimizes towards Root Mean Squared Error (RMSE). Many other works evaluate on a handful of somewhat randomly picked time series and then show plots of forecasts versus actuals as “proof” of how well their method works, without considering simple benchmarks or meaningful error measures, and other similar problems. Also, frequently forecasting competitions and research works introduce new evaluation measures and methodologies, sometimes neglecting the prior research, e.g., by seemingly not understanding that dividing a series by its mean will not solve scaling issues for many types of non-stationarities (e.g., strong trends). Thus, there is no generally accepted standard for forecast evaluation in every possible scenario. This gap has harmed the evaluation practices used along with ML methods for forecasting significantly in the past. It is damaging the area currently, with spurious results in many papers, with researchers new to the field not being able to distinguish between methods that work and methods that don’t, and the associated waste of resources.

Overall, this article makes an effort in the direction of raising awareness among ML practitioners regarding the best practices and pitfalls associated with the different steps of the point forecast evaluation process. Similar exhaustive efforts have been taken in the literature to review, formally define and categorize other important concepts in the ML domain such as concept drift (Webb et al. 2016), concept drift adaptation (Gama et al. 2014) and mining statistically sound patterns from data (Hämäläinen and Webb 2019). In the time series space, less comprehensive and/or systematic works in the direction of certain aspects of our work exist. Cerqueira et al. (2020) have performed empirical studies using different data partitioning and performance estimation methods on some real-world and synthetic datasets and presented guidelines around which methods work under different characteristics of time series. In the work by Petropoulos (2022) as well, those authors have a section dedicated to explaining forecast evaluation measures, best practices for both point and probabilistic forecasting as well as benchmarking. Ditzler et al. (2015) have conducted a survey on existing methods for learning in non-stationary environments and the associated difficulties and challenges. In the work by Shcherbakov et al. (2013), those authors have presented a review on several error measures for forecast evaluation along with their drawbacks and also proposed another new measure to specifically become robust to outliers on time series. Recommendations have also been given around selecting error measures under a specific context. Gujarati (2021) has provided a comprehensive overview on recent developments in econometric techniques in general using many examples.

The rest of this paper is structured as follows. Section 2 first introduces terminology associated with forecast evaluation, including different forms of non-stationarities/non-normality seen in time series data. Next, Sect. 3 details the motivation for this article, along with common pitfalls seen in the literature related to using sufficient datasets, selecting appropriate measures for evaluation, using competitive benchmarks, visualisation of results using forecast plots and data leakage in forecast evaluation. Then, in Sect. 4, we provide best practices and guidelines around different aspects of forecast evaluation including how to best partition the data for a given forecasting problem with non-stationarities involved with the series, how to select evaluation measures depending on the characteristics of the time series under consideration and details of popular techniques used for statistical testing for significance of differences between models. Finally, Sect. 5 concludes the paper by summarising the overall findings of the paper and highlighting the best practices for forecast evaluation. The code used for this work is publicly available for reproducibility of the results.^1^

## Terminology of forecast evaluation

**Fig. 1:**
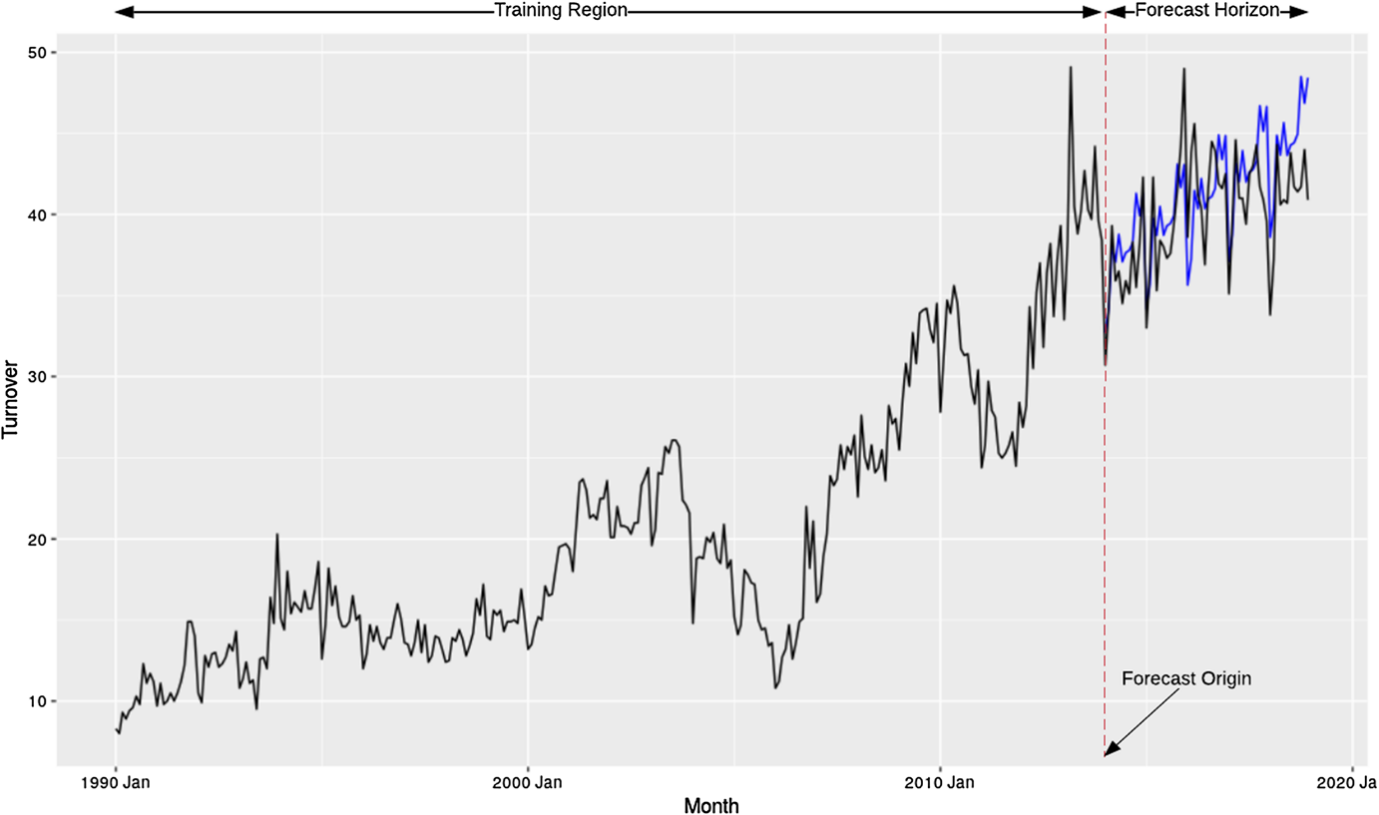
A forecasting scenario with training region of the data, forecast origin and the forecast horizon

This article focuses on point forecast evaluation, where the interest is to evaluate one particular statistic (mean/median) of the overall forecast distribution. However, we note that there are many works in the literature around predicting distributions and evaluating accordingly. Figure 1 indicates a common forecasting scenario with the training region of the data, the forecast origin which is the last known data point from which the forecasting begins and the forecast horizon. In this section we provide a general overview of the terminology used in the context of forecast evaluation.

**Fig. 2:**
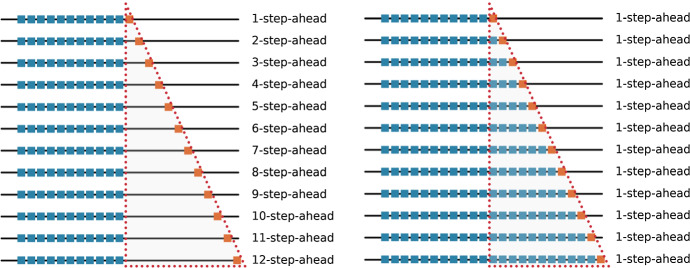
Comparison of fixed origin versus rolling origin setups. The blue and orange data points represent the training and testing sets respectively at each evaluation. The figure on the left side shows the fixed origin setup where the forecast origin remains constant. The figure on the right shows the rolling origin setup where the forecast origin rolls forward and the forecast horizon is constant. The red dotted lined triangle encloses all the time steps used for testing across all the evaluations. Compared to the fixed origin setup, it is seen that in the rolling origin setup, testing data instances in each evaluation pass on to the training set in the next evaluation step

In forecast evaluation, similar to other ML tasks, validation and test sets are used for hyperparameter tuning of the models and for testing. Evaluations on validation and test sets are often called *out-of-sample (OOS)* evaluations in forecasting. The two main setups for OOS evaluation in forecasting are *fixed origin evaluation* and *rolling origin evaluation* (Tashman 2000). Figure 2 shows the difference between the two setups. In the fixed origin setup, the forecast origin is fixed as well as the training region, and the forecasts are computed as one-step ahead or multi-step ahead depending on the requirements. In the rolling origin setup, the size of the forecast horizon is fixed, but the forecast origin changes over the time series (rolling origin), thus effectively creating multiple test periods for evaluation. With every new forecast origin, new data becomes available for the model which can be used for re-fitting of the model. The rolling origin setup is also called *time series cross-validation (tsCV)* and *prequential evaluation* in the literature (Hyndman and Athanasopoulos 2018; Gama et al. 2013).

Time series can have different forms of *non-stationarities* and *non-normality* and they make time series forecasting and evaluation a more difficult problem in comparison to other ML tasks. Listed below are some of such possibly problematic characteristics of time series.

1. Non-stationarities.
  Seasonality
  Trends (Deterministic, e.g., Linear/Exponential)
  Stochastic Trends / Unit Roots
  Heteroscedasticity
  Structural Breaks (sudden changes, often with level shifts)
2. Non-normality
  Non-symmetric distributions
  Fat tails
  Intermittency
  Outliers
3. Series with very short history

Non-stationarity in general means that the distribution of the data in the time series is not constant, but it changes depending on the time (see, e.g., Salles et al. 2019). What we refer to as non-stationarity in this work is the violation of strong stationarity defined as in Eq. (1) (Cox and Miller 1965). Strong stationarity is defined as the distribution of a finite window (sub-sequence) of a time series (discrete-time stochastic process) remaining the same as we shift the window across time. In Eq. (1), $y_{t}$ refers to the time series value at time step *t*; $\tau \in {\mathbb {Z}}$ is the size of the shift of the window and $n \in {\mathbb {N}}$ is the size of the window. $F_Y(y_{t+\tau }, y_{t+1+\tau }, ..., y_{t+n+\tau })$ refers to the cumulative distribution function of the joint distribution of $(y_{t+\tau }, y_{t+1+\tau }, ..., y_{t+n+\tau })$. Hence, according to Eq. (1), $F_Y$ is not a function of time, it does not depend on the shift of the window. In the rest of this paper, we refer to the violation of strong stationarity simply as non-stationarity.

1 $$\begin{aligned} F_Y(y_{t+\tau }, y_{t+1+\tau }, ..., y_{t+n+\tau }) = F_Y(y_{t}, y_{t+1}, ..., y_{t+n}), \hbox { for all}\,\, \tau \in {\mathbb {Z}}\,\, \hbox {and}\,\, n \in {\mathbb {N}}\nonumber \\ \end{aligned}$$

**Fig. 3:**
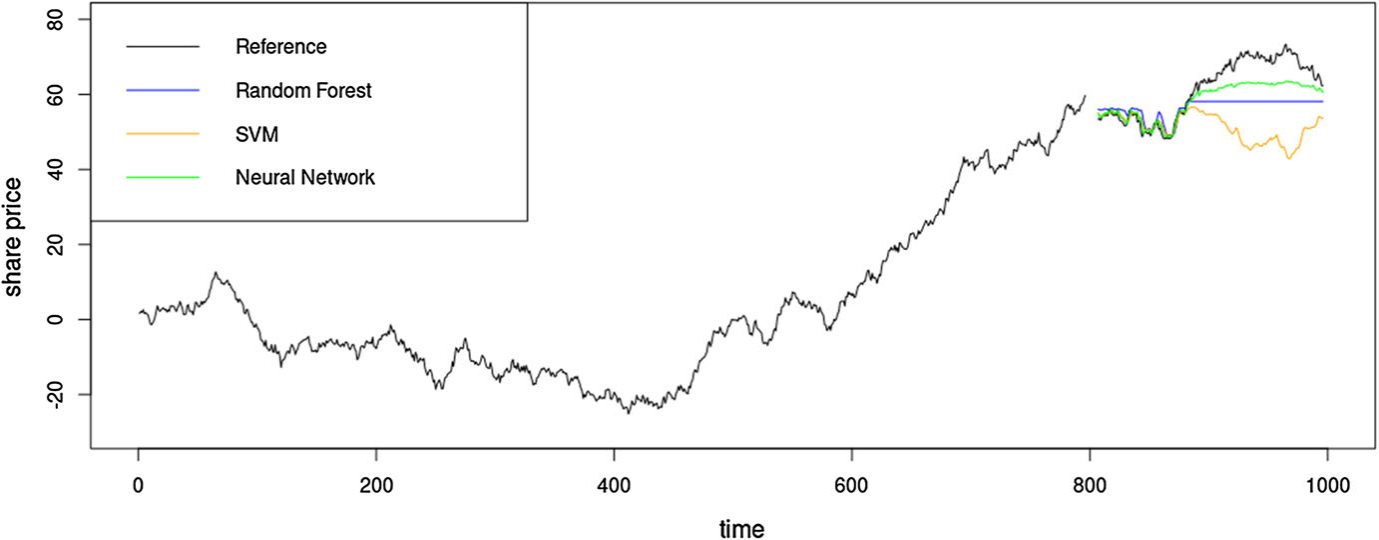
Forecasts from different models on a series with unit root based non-stationarity, with stochastic trends. In this example, we have a continuously increasing series (increasing mean) due to the unit root. The ML models are built as autoregressive models without any pre- or post-processing, and as such have very limited capacity to predict values beyond the domain of the training set, seen in the second part of the test set where predictions are considerably worse than in the first part

**Fig. 4:**
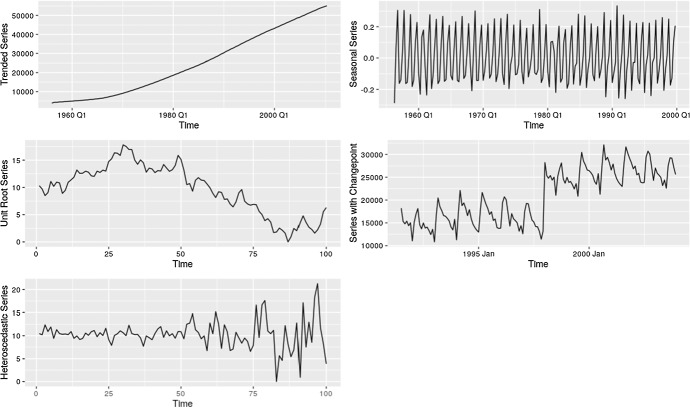
Different non-stationarities of series

Figure 3 gives an example of possible problems when building ML models on such data, where the models fail to produce reasonable forecasts as the range of values is different in the training and test sets. Different types of non-stationarities are illustrated in Fig. 4. *Seasonality* usually means that the mean of the series changes periodically over time, with a fixed length periodicity. Trends can be twofold; 1) *deterministic trends* - change the mean of the series 2) *stochastic trends* (resulting from unit roots) - change both the mean and variance of the series (Salles et al. 2019). Note that neither trend nor seasonality are concepts that have precise formal definitions. They are usually merely defined as smoothed versions of the time series, where for the seasonality the smoothing occurs over particular seasons (e.g., in a daily series, the series of all Mondays needs to be smooth, etc.). *Heteroscedasticity* changes the variance of the series and *structural breaks* can change the mean or other properties of the series. *Structural break* is a term used in Econometrics and Statistics in a time series context to describe a sudden change at a certain point in the series. It therewith has considerable overlap with the notion of *sudden concept drift* in an ML environment, where a sudden change of the data distribution is observed (Webb et al. 2016).

On the other hand, data can be far from normality, for example having fat tails, or when conditions such as outliers or intermittency are observed in the series. Non-stationarities and non-normality are both seen quite commonly in many real-world time series and the decisions taken during forecast evaluation depend on which of these characteristics the series have. There is no single universal rule that applies to every scenario.

## Motivation and common pitfalls

As briefly explained in Sect. 1, there exist many ML based papers for forecasting in the recent literature that are flawed or at least weak with regards to forecast evaluation. This section is devoted to provide the motivation of our work by discussing the most common problems and pitfalls associated with forecast evaluation in many recent literature.

### Benchmarks for forecast evaluation

Benchmarks are an important part of forecast evaluation. Comparison against the right benchmarks and especially the simpler ones is essential. However, often in the forecasting literature, newly proposed algorithms are not rigorously compared against the relevant benchmarks.

#### Naïve benchmark on finance series

**Fig. 5:**
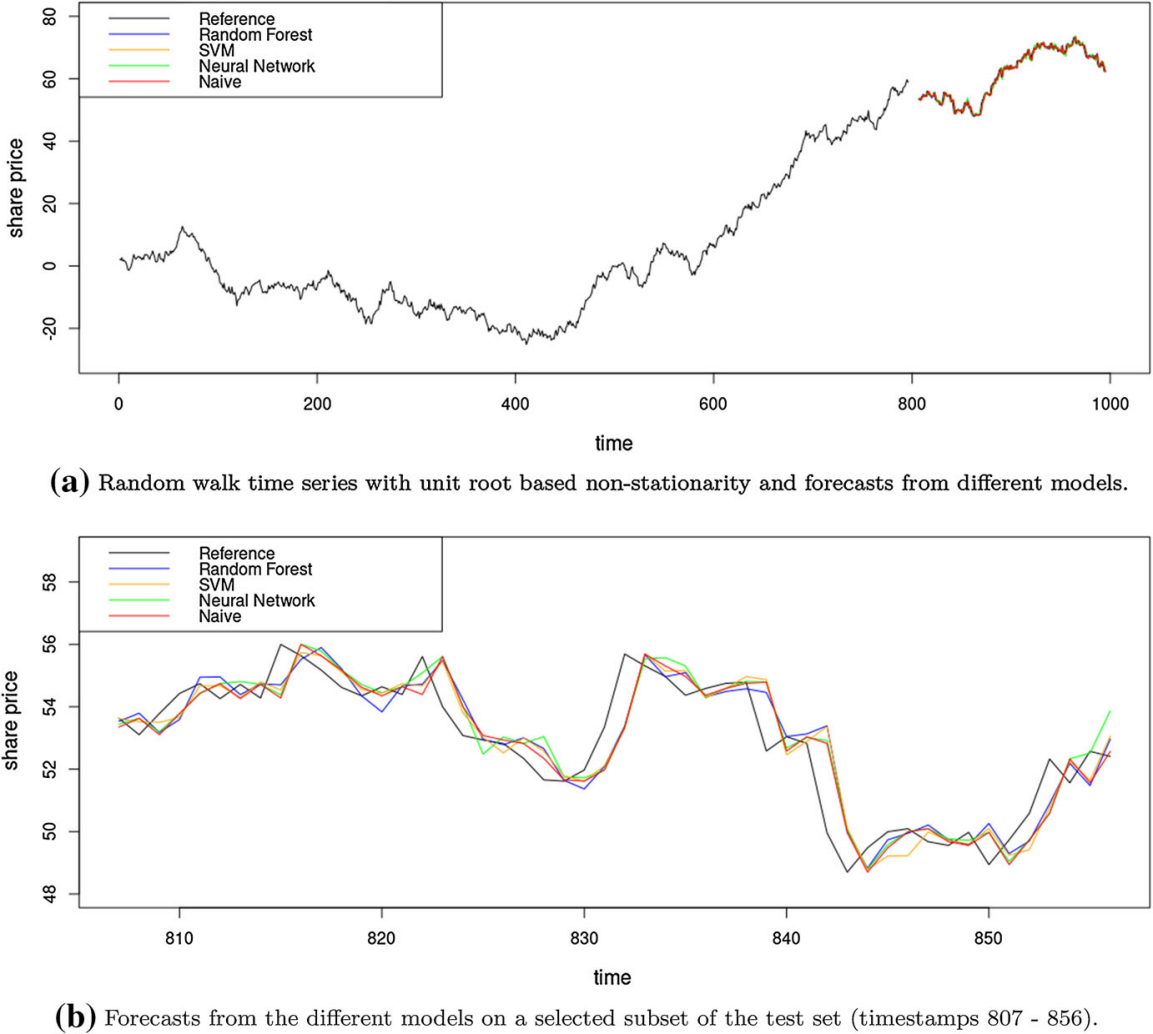
Forecasts from different models on a series with unit root based non-stationarity, with stochastic trends. The ML models are built as autoregressive integrated models, i.e., differencing has been done as pre-processing. The methods show very similar behaviour to the naïve forecast, and do not add any value over it by definition of the Data Generating Process (DGP) used

Arguably the simplest benchmark that is commonly employed in forecasting is the naïve forecast, also called persistence model or no-change model, that simply uses the last known observation as the forecast. It has demonstrated competitive performance in many scenarios (Armstrong Jan 2001), especially on series that demonstrate random walk properties. Equation (2) shows the definition of a random walk, where $\epsilon _t$ is white noise; i.e. sampled from a normal distribution. Accordingly, the naïve forecast at any time step in the horizon can be defined as in Eq. (3). As the naïve forecast is the last known observation, the forecast is a shifted version of the time series where the forecast simply follows the actuals (see Fig. 5b).

2 $$\begin{aligned}&y_{t+1} = y_{t} + \epsilon _t \end{aligned}$$

3 $$\begin{aligned}&{\hat{y}}_{t+h} = y_t \end{aligned}$$

Figure 5 illustrates the behaviour of different models that have been trained with differencing as appropriate preprocessing on a series that has a unit root based non-stationarity. If the series has no further predictable properties above the unit root (as in this example), i.e., it is a random walk where the innovation added to the last observation follows a normal distribution with a mean of zero, the naïve forecast is the theoretically best forecast, as also suggested by the RMSE values reported in Table 1. Other, more complex forecasting methods in this scenario will have no true predictive power beyond the naïve method, and any potential superiority, e.g., in error evaluations is by pure chance, and should be able to be identified as a spurious result on sufficiently large datasets.

**Table 1:** RMSE values of several ML methods and the naïve forecast on a random walk simulated time series using rolling origin data partitioning

Model	RMSE
Random forest (RF)	1.01
Support vector machine	1.00
Neural network	0.98
Naïve	0.96

The naïve forecast is the theoretically best forecasting method here

In many practical applications, we find series that show strongly integrated behaviour and therewith are close to random walks, as their innovations have high degrees of noise (such as stock market data, exchange rate data, and to a lesser extent wind power, wind speed). Here, a naïve forecast is a trivial yet competitive benchmark and without comparing against it, quality of more complex models cannot be meaningfully assessed. More complex methods will in such series usually show a behaviour where they mostly follow the series in the same way as the naïve forecast, and improvements are often small percentages over the performance of the naïve benchmark.

Financial time series such as exchange rates and stock prices are particularly problematic to forecast. For example, exchange rates are a function of current economic conditions and expectations about future valuations. Simultaneously, currencies are traded on the futures market (e.g., a market participant says they will buy X amount of US dollars in 1 year price for Y amount of Australian dollars), providing a market expectation of future price movements. The survey by Rossi (2013) has analysed the literature on exchange rate forecasting based on additional economic information and concluded that the most challenging benchmark is the random walk without drift model. Yet, ML based researchers have continued to introduce sophisticated Neural Network (NN) models for exchange rate forecasting without proper benchmarking. In the work by Wu et al. (2021), those authors have introduced a transformer based model with an embedded decomposition block and an autocorrelation mechanism to address long-term time series properties, called Autoformer. Their evaluation setup includes an exchange rate dataset used in many recent papers of this type (Lai et al. 2018), to be forecasted 720 days into the future. Predicting daily exchange rates based on only past exchange rates nearly 2 years into the future may sound like an outrageous claim to Economists already, and those authors themselves state in that paper, that the dataset contains no obvious periodicities and thus is hard to be predicted compared to other datasets. It is thus unclear how the decomposition mechanism used in their proposed model should in any way make a valid contribution to predicting these series. As those authors have not compared their model against the naïve benchmark, we experiment using a naïve forecast on this exchange rate dataset, under the same evaluation setup as those authors. The results are as reported in Table 3. Table 3 reports the results for Autoformer both from our experiments as well as the experiments reported in the paper. As seen here, the error values that we get for Autoformer are slightly different from the error values reported in the paper, due to the randomness of the seed values used in the experiments. Regardless, the naïve forecast beats both the results from Autoformer across all the horizon sizes tested by a considerable margin, indicating that the proposed method (and all comparison methods used in the original paper) is essentially useless on this particular dataset. Also keep in mind that in this example Autoformer takes hours to run on CPU or alternatively needs a GPU with 24GB of memory, to finally arrive at results that are worse than trivial results that require essentially no computation at all.

**Table 2:** Results from several reruns of the FiLM model in the work by Zhou et al. (2022a)

Trial No.	MSE	MAE
1	1.100	0.798
2	1.302	0.869
3	1.491	0.940
4	1.218	0.841
5	1.261	0.855

**Table 3:** Results from the naïve forecast and the Autoformer model on the exchange rate dataset

Horizon	Naïve		Autoformer (Rerun)		Autoformer (Original Paper)	
	MAE	MSE	MAE	MSE	MAE	MSE
96	**0.192**	**0.078**	0.279	0.149	0.323	0.197
192	**0.282**	**0.158**	0.399	0.299	0.369	0.300
336	**0.388**	**0.287**	0.504	0.460	0.524	0.509
720	**0.694**	**0.817**	0.963	1.552	0.941	1.447

Best models shown in boldface font

More recently, in the work by Zhou et al. (2022a), those authors have proposed a Frequency improved Legendre Memory (FiLM) model which helps with removing noisiness in signals and also preserves historical information for long-term forecasting. In that paper too, those authors have experimented on the same exchange rate dataset. According to the results reported in that paper, that model outperforms the naïve forecast on the longest horizon size of 720 on the multivariate forecasting study of the exchange rate dataset (the FiLM model has reported an MSE and MAE of 0.727 and 0.669, respectively, whereas the naïve forecast has an MSE of 0.817 and an MAE of 0.694 as reported in Table 3). We have attempted to reproduce the same results of the FiLM model, to investigate the statistical significance of the difference compared to the naïve forecast. However, using five trials we have been unable to reproduce the exact same results on the horizon size of 720, and all the results we have been able to obtain using the code published with the original paper show a performance inferior to the naïve forecast. These results from the five trials are reported in Table 2. Also note that within each trial, the experiment is repeated 5 times using random seeds and the mean of the metrics is reported to comply with what the authors have done in their work. As reported in Table 2, the inability of the FiLM model to consistently outperform the naïve forecast indicates that the results reported in the paper are most likely spurious and obtained randomly by chance.

There have been many other recent works both published in ML outlets or published on preprint servers alone which have followed similar approaches to introduce NN based algorithms for long-term forecasting and then tested them using the same exchange rate dataset but without comparisons against the naïve benchmark and therewith leading to the same problematic conclusions of superiority of the respective methods, namely Zhou et al. (2022b); Challu et al. (2022); Du et al. (2022); Sun and Boning (2022); Woo et al. (2022); Zhou et al. (2022c); Li et al. (2022a); Shabani et al. (2022). While it is good practice to follow a common setup for further research which allows for comparison against the previous state-of-the-art methods, if the original setup is flawed this means that all successors are flawed as well. As such, the benchmarks and the error measure used play an important role in such a setting. For instance, by using a relative error measure (detailed further in Sect. 4.2) that lets us directly compare against a simple benchmark such as the naïve, we can be certain of the competitiveness of the model against simple methods.

Apart from the failure to use the correct benchmarks as explained above, there are further issues associated with these exchange rate time series, that makes producing forecasts for them a fundamentally flawed task. One issue is that exchange rate data (and in particular this dataset) is based on trading days, meaning that the time series that all the aforementioned works have dealt with do not contain weekends and are not equally spaced, so that any comments on seasonality and cycle length in these papers are likely wrong. However, the most important point is that data is more than input into an algorithm. The large body of literature in economics and finance over 50 years states that it is not sensible to forecast exchange rate time series, as it violates the efficient market hypothesis (Fama 1970). The nature of a market is that the price reflects all the information publicly available, and even if it does not do it for a short period (such as minutes or days; or milliseconds in high-frequency trading), and some investors enjoy extra information, they will act on it, and the market price will adapt. There is a known persistence in the return volatility of foreign exchange rate markets (Berger et al. 2009). Still, there is no evidence that it is reasonable to assume to forecast exchange rates 720 days into the future. The final open question of forecasting these exchange rates completely left out by the aforementioned literature is, why we are forecasting exchange rate in the first place. Is the intention to trade on that information, or is it for risk management? How does an error measure that translates to being more than 50% of the time wrong lead to anything else than the bankruptcy of the user? Would the authors themselves be satisfied that their pension fund is using their own model for investing their money? We guess it is fair to answer this with no.

Similar considerations hold for stock price forecasting. Some examples from the recent ML literature in this area that benchmark on stock market related data without comparisons against the naïve benchmark are Shen et al. (2020); Du et al. (2021); Lin et al. (2021). Stock market data is another classical example where data is abundant, but stock returns are deemed to be “almost unpredictable” (Engle 2003), especially using only past stock prices as inputs alone, in the classic Economics literature, as stock prices are again assumed to not be a function of their own past but of current market conditions and expectations about future valuations, and in an efficient market, forecasting using only past stock price data will not yield results more accurate than a naïve forecast. It is important to note in this context that this holds for stock prices and returns, but not volatility, which is predictable, e.g., using autoregressive conditional heteroskedasticity (ARCH), a finding which led to the award of the 2003 Nobel Memorial Prize in Economic Sciences to Robert F. Engle (Engle 2003).

As such, papers that claim that they can predict stock prices or returns, or exchange rates based on historic readings of these same signals alone need to be aware that their claims contradict some central notions in Economics and that they need to be evaluated very rigorously, as their results are likely to be spurious.

#### Other simple forecasting benchmarks

On series that have clear seasonal patterns, models should accordingly be benchmarked against the seasonal naïve model as the most simplistic benchmark, and also other simple benchmarks are commonly used in forecasting. In the work by Zhou et al. (2021) those authors have proposed a novel memory and time efficient transformer based architecture, namely Informer for long sequence forecasting. That paper has also won the outstanding paper award at the Association for the Advancement of Artificial Intelligence (AAAI) conference 2021. In that work several experiments have been conducted using Electricity Transformer Temperature data (ETT), Electricity Consumption Load (ECL)^2^ data and Weather data. The ETT and ECL hourly datasets clearly show strong multiple seasonal patterns (being hourly series, daily, weekly, and yearly patterns are to be expected). However, the Informer model has only been benchmarked against non-seasonal ARIMA which is not capable of handling multiple seasonalities, and is a grotesquely misspecified model that would not be used in practice. To claim its superior performance in the long horizon forecasting problems, the proposed Informer model in this case needs to be compared against statistical standard benchmarks that inherently handle multiple seasonalities well, such as the Dynamic Harmonic Regression ARIMA (DHR-ARIMA) model and the TBATS model (Hyndman and Athanasopoulos 2018). To demonstrate this, we conduct an experiment with a DHR-ARIMA model on the ETTh$_{1}$ and the ECL datasets on their respective longest horizon sizes (720 for the ETTh$_{1}$ dataset and 960 for the ECL dataset) for the univariate forecasting task. For the ETTh$_{1}$ dataset, daily and yearly seasonal patterns are incorporated where as for the ECL dataset, all daily, weekly and yearly seasonalities are included using Fourier terms in the DHR-ARIMA model. The results are reported in Table 4, along with the results for the benchmark models shown in the original paper. The horizon size is shown within parentheses next to the dataset name in Table 4. As seen from these results, when the Fourier terms are incorporated to capture the multiple seasonalities, the standard DHR-ARIMA can outperform ARIMA as well as the two variants of the proposed algorithm, Informer and Informer$^\dagger$.

**Table 4:** Results of the DHR-ARIMA model along with Informer and the other benchmarks on the univariate forecasting task in the work by Zhou et al. (2021)

Model	ETTh$_{1}$(720)		ECL(960)	
	MSE	MAE	MSE	MAE
Informer	0.269	0.435	0.582	0.608
Informer$^\dagger$	0.257	0.421	0.594	0.638
LongTrans	0.273	0.463	0.624	0.645
Reformer	2.112	1.436	7.019	5.105
LSTMa	0.683	0.768	1.545	1.006
DeepAR	0.658	0.707	0.657	0.683
ARIMA	0.659	0.766	1.370	0.982
Prophet	2.735	3.253	6.901	4.264
**DHR-ARIMA**	**0.140**	**0.297**	**0.433**	**0.499**

$\dagger$ which uses a canonical self-attention mechanism

Best models shown in boldface font

Apart from that, the recent work by Zeng et al. (2022) has challenged the long-term time series forecasting capability of transformer based models in general by comparing against a relatively simple linear layer based NN, i.e., a set of linear models trained for the forecasting horizon in question directly. As those authors have stated in their work, most of the performance gains of the aforementioned transformer based models for long-term forecasting are due to comparing their direct multi-step ahead forecasts against iterative forecasts that are produced from more traditional methods, which inherently have error accumulation issues due to the recursive nature of forecasting. This claim once again emphasises the need to perform comparisons with the right and the most competitive established forecasting benchmarks for the relevant study, as directly trained linear models have been shown to outperform all the considered transformer architectures in that work.

### Datasets for empirical evaluations

Another common problem in the ML based forecasting literature is that many works do not use sufficient amounts of datasets/time series for the experiments for reasonably claiming the superior performance of the proposed algorithms. While it may be somewhat subjective what amount of series is sufficient, oftentimes papers use only a handful of series when the authors clearly don’t seem to care about their particular application and/or when hundreds of series could be readily available for the same application case, e.g., in notorious stock return prediction tasks. Some examples along these lines (there are many more in the literature) are the works of Liu et al. (2021, 2020); Godfrey and Gashler (2018); Shen et al. (2020), and Zhang et al. (2021). In particular, Zhang et al. (2021) use 3 time series in total, a simulated AR(1) process, a bitcoin price series and an influenza-like illness series, to evaluate their non-parametric neural network method. While the influenza-like illness series may be a good forecasting case study, basically the same considerations as for exchange rates and stock prices hold for bitcoin prices, though bitcoin was presumably a less efficient market, especially in its infancy. The best model to forecast an AR(1) process is trivially an AR(1) model (which is not used as a benchmark in that paper), so fitting complex neural networks to this series makes very limited sense.^3^ The authors are here effectively fitting a neural network to model a 2-dimensional linear relationship plus noise.

### Evaluation measures for forecasting

A variety of evaluation measures have been proposed for forecast evaluation over the years, and thus ML based forecasting researchers seem to be in a situation unable to clearly pick the evaluation measures that best suit their requirements and the data at hand. For example, in the work by Lai et al. (2018), those authors have used two measures Root Relative Squared Error (RSE) and Empirical Correlation Coefficient (CORR) for the evaluation which both use scaling based on the mean of the time series. While this may work as a scaling technique for time series that have minimal or no trends, for series that contain trend based non-stationarities this does not scale the series meaningfully. Yet, this information is only implicit and not conveyed to the reader in their work. Consequently, there have been many other works which followed the same evaluation setup and the measures without any attention to whether the used series contain trends or not (examples are Guo et al. 2022; Shih et al. 2019; Wu et al. 2020; Ye et al. 2022). Although this allows for direct comparisons against previous work, it also has caused all successive works to overlook the same issues with trended time series with the used error measures.

Some works also use scale-dependent measures such as Mean Squared Error (MSE), RMSE and MAE on multivariate datasets having many time series (examples are Cui et al. 2021; Du et al. 2021; Ye et al. 2022). While this is reasonable if all the series in the dataset have similar scales, if the scales are different, this means that the overall error value would be driven by particular series. Some have used the coefficient of determination (R$^2$) between the forecasts and the actual values as a forecast evaluation measure as well (for example Shen et al. 2020; Zhou et al. 2022). This can be a quite misleading evaluation measure especially in the case of random walk time series, which may give almost perfect R$^2$ values (close to 1) due to the following nature of the series indicating a competitive performance of the model, whereas in reality the series does not have any predictable patterns at all. MAPE is another evaluation measure commonly applied incorrectly on series having very small values in the range [−1, 1] (examples are Moon et al. 2022; Wu et al. 2020). Due to the denominator of the MAPE which is the actual value of the time series, on series having values close to 0, MAPE gives excessively large values irrespective of the actual prediction.

### Forecast plots

Plots with time series forecasting results can be quite misleading and should be used with caution. Analysing plots of forecasts from different models along with the actuals and concluding that they seem to fit well can lead to wrong conclusions. It is important to use benchmarks and evaluation metrics that are right for the context. In a scenario like a random walk series as in Fig. 5 as stated before, visually our models may look like achieving similar or better accuracy than the naïve method, but it will be a spurious result. The visual appeal of a generated forecast or the possibility of such a forecast to happen in general are not good criteria to judge forecasts. However, many recent forecasting literature seem to use forecast plots that do not convey much information regarding the performance of the methods (for example Liu et al. 2021, 2020; Du et al. 2021).

**Fig. 6:**
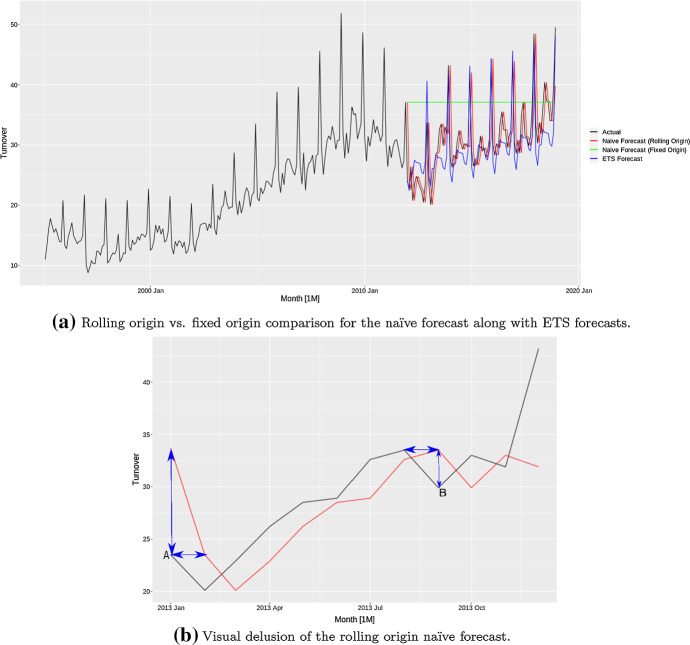
Properties of the naïve forecast

Figure 6a shows a monthly time series with yearly seasonal patterns along with forecasts from the ETS model. The figure furthermore shows the forecasts under fixed origin and rolling origin data partitioning schemes for the naïve forecast. When periodic re-fitting is done with new data coming in as in a rolling origin setup, the naïve forecast gets continuously updated with the last observed value. For the fixed origin context on the other hand, the naïve forecast remains constant as a straight line corresponding to the last seen observation in the training series. We see that with a rolling-origin naïve forecast, the predictions tend to look visually very appealing, as the forecasts follow the actuals and our eyes are deceived by the smaller horizontal distances instead of the vertical distances that are relevant for evaluation. Figure 6b illustrates this behaviour. It is clear how the horizontal distance between the actuals and the naïve forecast at both points A and B are much less compared to the vertical distances which are the relevant ones for evaluation. In these situations we need to rely on the error measures, as the plots do not give us much information. As reported in Table 5, for this scenario the ETS forecasts have a smaller RMSE error compared to both rolling origin and fixed origin naïve forecasts.

**Table 5:** RMSE values of rolling origin versus fixed origin naïve forecasts and ETS forecasts on the time series in Fig. 6a

Model	RMSE
Naïve (Rolling Origin)	31.23
Naïve (Fixed Origin)	37.10
ETS	29.93

**Table 6:** RMSE values of several methods and the naïve forecast on a unit root based time series using fixed origin data partitioning

Model	RMSE
RF	3.18
Support Vector Machine	17.88
Neural Network	35.22
ARIMA	6.44
Naïve	3.04

**Fig. 7:**
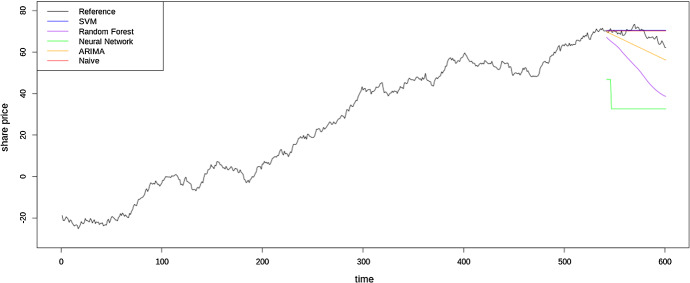
Fixed origin forecasts from several models and the naïve forecast on a random walk time series

Figure 7 shows another series having unit root based non-stationarity and fixed origin forecasts from several models and the naïve forecast for a forecast horizon of 60 time steps ahead. This shows another issue with using plots to determine forecast accuracy. As explained previously, on a random walk time series, a naïve forecast is the theoretically best forecast that can be obtained. This is also clarified by the RMSE values for these forecasts from the different models as reported in Table 6. However, the naïve forecast for fixed origin is a constant. Although this does not look realistic, and in most application domains we can be certain that the actuals will not be constant, practitioners may mistakenly identify such behaviour as a potential problem with the models, where this forecast is indeed the best possible forecast in the sense that it minimizes the error based on the information available at present.

In summary, plots of the forecasts can be deceiving and should be used mostly for sanity checking. Decisions should mostly be made based on evaluations with error measures and not based on plots.

### Data leakage in forecast evaluation

Data leakage refers to the inadvertent use of data from the test set, or more generally data not available during inference, while training a model. It is always a potential problem in any ML task. For example, Kaufman et al. (2012) present an extensive review on the concept of data leakage for data mining and potential ways to avoid it. Arnott et al. (2019) discuss this in relation to the domain of finance. Hannun et al. (2021) propose a technique based on Fisher information that can be used to detect data leakage of a model with respect to various subsets of the dataset. Brownlee (2020) also provide a tutorial overview on data preparation for common ML applications while avoiding data leakage in the process. However, in forecasting data leakage can happen easier and can be harder to avoid than in other ML tasks such as classification/regression.

Forecasting is usually performed in a self-supervised manner with rolling origin evaluations where periodic re-training of models is performed, and within this re-training, it is normal that data travels from the test to the training set as seen on Fig. 2. As such, it is often difficult and not practical to separate training and evaluation code bases. As such, we often have to trust the software provider that everything is implemented correctly, and an external evaluation is difficult.

**Fig. 8:**
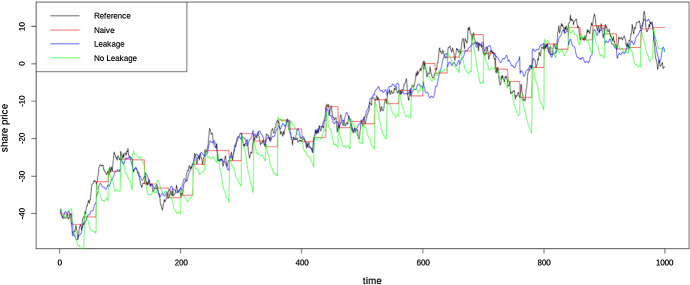
Forecasts from a model with leakage and no leakage on a time series having unit root based non-stationarity

Also, more indirect forms of data leakage can happen in forecasting. In analogy to classification/regression, where data leakage sometimes happens by normalising data before partitioning for cross-validation, in forecasting, data leakage can happen by performing smoothing, decomposition (mode decomposition), normalisation etc. over the whole series before partitioning for training and testing. This can sometimes be seen in ML based forecasting literature. In the work by Ran et al. (2023) those authors perform Empirical Mode Decomposition (EMD) presumably over the whole series. Zhou et al. (2022) perform normalisation of the time series presumably before the train, test set split. Kuranga and Pillay (2022) also perform 0-1 normalisation of the time series presumably before modelling. They have used two forecast horizons on the same series (in a rolling origin fashion), yet there is no mention to performing the normalisation twice to reflect the training data in each case. Hence that could lead to data leakage too. To demonstrate the effect of leakage, we perform an experiment on a random walk time series using a rolling origin setup. EMD is performed on the series and the intrinsic mode functions are each modelled using Random Forest (RF) models and the residue modelled using an ARIMA model. In the data leakage scenario, EMD is performed on the whole series whereas in the no leakage scenario, EMD is performed iteratively for each new training set in the rolling origin setup. As EMD has some low-frequent components, applying it over the full series, these low-frequent components contain considerable information about the series’ future. The forecast horizon is set to 20 steps with 50 rolling origins constituting an overall test set of 1000 steps. The results are reported in Table 7. As seen by the results, with data leakage the model becomes the best model even outperforming the naïve forecast which is the theoretically best forecast on a random walk series. The *p*-value indicates the *p*-value from the Wilcoxon signed-rank test applied to measure the statistical significance of the differences of the two techniques (leakage vs. no leakage) against the naïve forecast. A *p*-value $<0.05$ indicates that the method is significantly different from the naïve forecast. Therefore, from these results it is clear that although when no leakage is present in the method, the forecast is significantly worse than the naïve forecast, when leakage is incorporated, the method has a nearly significant *p*-value rendering it better than the naïve forecast. These forecasts are further visualized in the plots in Fig. 8. Data leakage can happen even when extracting features such as tsfeatures (Hyndman et al. 2019), catch22 (Lubba et al. 2019) that are not constant over time, to feed as inputs to the model. Thus, features can be extracted only from the training set data, and may need to be re-calculated either periodically or over the specific input windows. However, this can be computationally expensive.

**Table 7:** RMSE values for the leakage and no leakage experiments on a unit root based time series

Model	RMSE	*p*-value
Naïve	3.46	–
Leakage Model	3.12	0.067
No Leakage Model	5.65	1.85 x 10 $^{-6}$

The *p*-values from the statistical tests of differences against the naïve forecast are also reported

Another type of leakage especially when training global models that learn across series, which is common practice nowadays for ML models, is when one series in the dataset contains information about the future of another series. For example with an external shock like COVID-19 or a global economy collapse, all the series in the dataset can be equally affected. Therefore, if the series in the dataset are not aligned and one series contains the future values with respect to another, when splitting the training region, future information can be already included within the training set. However, in real world application series are usually aligned so that this is not a big problem. On the other hand, in a competition setup such as the M3 and M4 forecasting competitions (Makridakis and Hibon 2000; Makridakis et al. 2020), where the series are not aligned, this can easily happen (Talagala 2020).

Data leakage can also happen simply due to using the wrong forecast horizon. This can happen by using data that in practice will become available later. For example, we could build a one-day-ahead model, but use summary statistics over the whole day. This means that we cannot run the model until midnight, when we have all data from that day available. If the relevant people who use the forecasts work only from 9am-5pm, it becomes effectively a same-day model. The other option is to set the day to start and end at 5pm everyday, but that may lead to other problems.

In conclusion, data leakage dangers are common in self-supervised forecasting tasks. It is important to avoid leakage problems 1) in rolling origin schemes by being able to verify and trust the implementation, as external evaluation can be difficult 2) during preprocessing of the data (normalising, smoothing etc.) and extracting features such as tsfeatures by splitting the data into training and test sets beforehand 3) by making sure that within a set of series, one series does not contain in its training period potential information about the future of another series.

## Guidelines and best practices for forecast evaluation

Forecast model building and evaluation typically encompasses the following steps.

- Data partitioning
- Forecasting
- Error Calculation
- Error Measure Calculation
- Statistical Tests for Significance (optional)

The process of evaluation in a usual regression problem is quite straightforward. The best model out of a pool of fitted models is selected based on the value of a final error measure on the validation set. The relevant error measures used etc. are standard and established as best practices in these domains. However, when it comes to forecast evaluation, many different options are available for each of the aforementioned steps and no standards have been established thus far, and hence all the pitfalls in the literature as explained in Sect. 3. Therefore, in this section we are presenting a set of best practices and guidelines for each of the aforementioned steps in forecast evaluation.

### Data partitioning

In the following we present the guidelines around data partitioning for forecast evaluation.

#### Fixed origin setup

Fixed origin setup is a faster and easier to implement evaluation setup. However, with a single series, the fixed origin setup only provides one forecast per each forecast step in the horizon. According to Tashman (2000), a preferred characteristic of OOS forecast evaluation is to have sufficient forecasts at each forecast step. Also, having multiple forecasts for the same forecast step allows to produce a forecast distribution per each step for further analysis. Another requirement of OOS forecast evaluation is to make the forecast error measures insensitive to specific phases of business (Tashman 2000). However, with a fixed origin setup, the errors may be the result of particular patterns only observable in that particular region of the horizon (Tashman 2000). Therefore, the following multi period evaluation setups are introduced as opposed to the fixed origin setup.

#### Rolling origin, time series cross-validation and prequential evaluation setups

Armstrong and Grohman (1972) are among the first researchers to give a descriptive explanation of the rolling origin evaluation setup. Although the terms rolling origin setup and tsCV are used interchangeably in the literature, in addition to the forecast origin rolling forward, tsCV also allows to skip origins, effectively rolling forward by more than one step at a time (analogously to the difference between a leave-one-out CV and a k-fold CV).

With such multi period evaluations, each time the forecast origin updates, the model encounters new actual data. With new data becoming available, we have the options to – in the terminology of Tashman (2000) – either update the model (feed in new data as inputs) or recalibrate it (refit with new data). Although for some of the traditional models such as ETS and ARIMA, the usual practice (and the implementation in the forecast package) in a rolling origin setup is to recalibrate the models, for general ML models it is more common to mostly just accept new data as inputs and only periodically retrain the model (updating). As ML methods tend to work better with higher granularities, re-fitting is not an option (for example, a monthly series predicted with ETS vs. a 5-minutely series predicted with Light Gradient Boosting Models). Therefore, retraining as the most recent data becomes available happens in ML methods mostly only when some sort of concept drift (change of the underlying data generating process) is encountered (Webb et al. 2016).

**Fig. 9:**
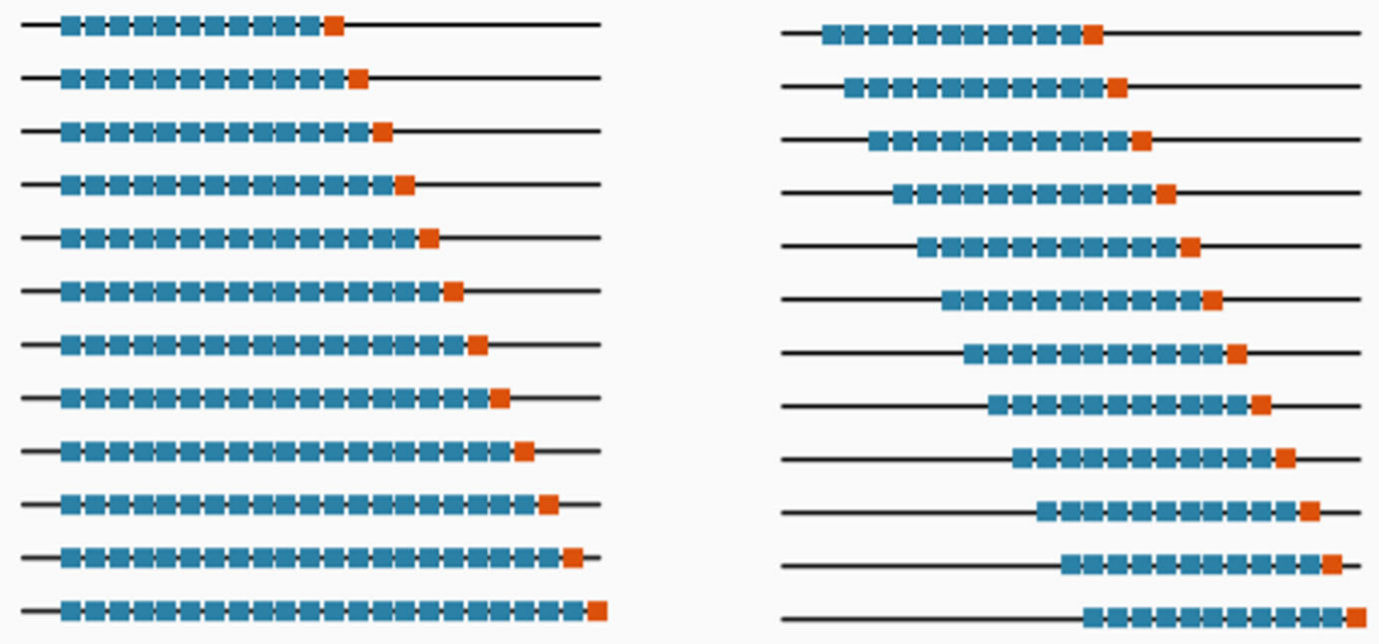
Comparison of Expanding Window versus Rolling Window setups. The blue and orange points represent the training and test sets, respectively. The figure on the left side shows the Expanding Window setup where the training set keeps expanding. The figure on the right shows the Rolling Window setup where the size of the training set keeps constant and the first point of the training set keeps rolling forward

Rolling origin evaluation can be conducted in two ways; 1) Expanding window setup 2) Rolling window setup. Figure 9 illustrates the difference between the two approaches. The expanding window method is a good setup for small datasets/short series (Bell and Smyl 2018). On the other hand, the rolling window setup removes the oldest data from training as new data becomes available (Cerqueira et al. 2020). This will not make a difference with forecasting techniques that only minimally attend to the distant past, such as ETS, but may be beneficial with pure autoregressive ML models, that have no notion of time beyond the windows. A potential problem of the rolling origin setup is that the first folds may not have much data available. However, the size of the first folds is not an issue when dealing with long series, thus making rolling origin setup a good choice with sufficient amounts of data. On the other hand, with short series it is also possible to perform a combination of the aforementioned two rolling origin setups where we start with an expanding window setup and then move to a rolling window setup.

#### (Randomized) Cross-validation

**Fig. 10:**
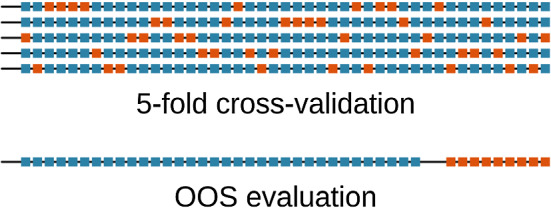
Comparison of randomized CV versus OOS evaluation. The blue and orange dots represent the training and test sets, respectively. In the usual k-fold-CV setup the testing instances are chosen randomly over the series. In OOS, the test set is always reserved from the end of the series

The aforementioned two techniques of data partitioning preserve the temporal order of the time series when splitting and using the data. A common misconception is that this is always a necessity when dealing with time series. Another form of data partitioning is to use a common randomized CV scheme as first proposed by Stone (1974). This scheme is visualized in Fig. 10. Compared to the aforementioned validation schemes which preserve the temporal order of the data, this form of randomized CV strategy can make efficient use of the data, since all the data is used for both model training as well as evaluation in iterations (Hastie et al. 2009). This helps to make a more informed estimation about the generalisation error of the model.

However, this form of random splitting of a time series does not preserve the temporal order of the data, and is therefore oftentimes not used and seen as problematic. The common points of criticism for this strategy are that, 1) it can make it difficult for a model to capture serial correlation between data points (autocorrelation) properly, 2) potential non-stationarities in time series can cause problems (for example, depending on the way that the data is partitioned, if all data from Sundays happen to be in the test set but not the training set in a series with weekly seasonality, then the model will not be able to produce accurate forecasts for Sundays since it has never seen data of Sundays before), 3) the training data contains future observations and the test set contains past data due to the random splitting and 4) since evaluation data is reserved randomly across the series, the forecasting problem shifts to a missing value imputation problem which certain time series models are not capable of handling (Petropoulos 2022).

Despite these problems, randomized CV can be applied to pure AR models without serial correlation issues. Bergmeir et al. (2018) theoretically and empirically show that CV performs well in a pure AR setup, as long as the models nest or approximate the true model, as then the errors are uncorrelated, leaving no dependency between the individual windows. To check this, it is important to estimate the serial correlation of residuals. For this, the Ljung-Box test (Ljung and Box 1978) can be used on the OOS residuals of the models. While for overfitting models there will be no autocorrelation left in the residuals, if the models are underfitted, some autocorrelation will be left in the OOS residuals. If there is autocorrelation left, then the model still does not use all the information available in the data, which means there will be dependencies between the separate windows. In such a scenario, CV of the time series dataset will not hold valid, and underestimate the true generalisation error. The existence of significant autocorrelations anyway means that the model should be improved to do better on the respective series (increase the AR order to capture autocorrelation etc.), since the model has not captured all the available information. Once the models are sufficiently competent in capturing the patterns of the series, for pure AR setups (without exogenous variables), standard k-fold CV is a valid strategy. Therefore, in situations with short series and small amounts of training data, where it is not practically feasible to apply the aforementioned tsCV techniques due to the initial folds involving very small lengths of the series, the standard CV method with some control of underfitting of the models is a better choice with efficient use of data.

The aforementioned problem that the testing windows can contain future observations, is also addressed by Bergmeir et al. (2018). With the CV strategy, the past observations not in the training data but existing in the test set can be considered missing observations, and the task is seen more as a missing value imputation problem rather than a forecasting problem. Many forecasting models such as ETS (in its implementation in the forecast package (Hyndman and Athanasopoulos 2018)), which iterate throughout the whole series, cannot properly deal with missing data. For Recurrent Neural Networks (RNN) as well, due to their internal states that are propagated forward along the series, standard k-fold CV which partitions data randomly across the series is usually not applicable. Therefore, for such models, the only feasible validation strategy is tsCV. Models such as ETS can anyway train competitively with minimal amounts of data (as is the case with the initial folds of the tsCV technique) and thus, are not quite problematic with tsCV. However, for reasonably trained pure AR models, where the forecasts for one window do not in any way depend on the information from other windows (due to not underfitting and having no internal state), it does not make a difference between filling the missing values in the middle of the series and predicting future values, where both are performed OOS. Nevertheless, the findings by Bergmeir et al. (2018) are restricted to only stationary series.

#### Data partitioning for non-stationary data

Cerqueira et al. (2020) experimented using non-stationary series, where they have concluded that OOS validation procedures preserving the temporal order (such as tsCV), are the right choice when non-stationarities exist in the series. However, a possible criticism of that work is the choice of models. We have seen in Sect. 3 that ML models are oftentimes not able to address certain types of non-stationarities out of the box. More generally speaking, ML models are non-parametric, data-driven models. As such, the models are typically very flexible and the function fitted depends heavily on the characteristics of the observed data. Though recently challenged (Balestriero et al. 2021), a common notion is that ML models are typically good at interpolation and lack extrapolation capabilities. The models used by Cerqueira et al. (2020) include several ML models such as a Rule-based Regression (RBR) model, a RF model and a Generalized Linear Model (GLM), without in any way explicitly tackling the non-stationarity in the data (similar to our example in Sect. 3). Thus, if a model is poor and not producing good forecasts, performing a validation to select hyperparameters, using any of the aforementioned CV strategies, will be of limited value. Furthermore, and more importantly, non-stationarity is a broad concept and both for the modelling and the evaluation it will depend on the type of non-stationarity which procedures will perform well. For example, with abrupt structural breaks and level shifts occurring in the unknown future, but not in the training and test set, it will be impossible for the models to address this change and none of the aforementioned evaluation strategies would do so either. In this situation, even tsCV would grossly underestimate the generalisation error. For a more gradual underlying change of the DGP, a validation set at the end of the series would be more appropriate since in that case, the data points closer to the end of the series may be already undergoing the change of the distribution. On the other hand, if the series has deterministic trend or seasonality, which are straightforward to forecast, they can be simply extracted from the series and predicted separately whereas the stationary remainder can be handled using the model. In such a setup, the k-fold CV scheme will work well for the model, since the remainder complies with the stationarity condition. For other non-deterministic trends, there are several data pre-processing steps mentioned in the literature such as lag-1 differencing, logarithmic transformation (for exponential trends), Seasonal and Trend Decomposition using Loess (STL Decomposition), local window normalisation (Hewamalage et al. 2021), moving average smoothing, percentage change transform, wavelet transform etc. (Salles et al. 2019). The findings of Salles et al. (2019) have concluded that there is no single universally best transformation technique across all datasets; rather it depends on the characteristics of the individual datasets. If appropriate data pre-processing steps are applied to enable models to handle non-stationarities, with a pure AR setup, the CV strategy still holds valid after the data transformation, if the transformation achieves stationarity. As such, to conclude, for non-stationarities, tsCV seems the most adequate as it preserves the temporal order in the data. However, there are situations where also tsCV will be misleading, and the forecasting practitioner will already for the modelling need to attempt to understand the type of non-stationarity they are dealing with. This information can subsequently be used for evaluation, which may render CV methods for stationary data applicable after transformations of the data to make them stationary.

#### Summary and guidelines for data partitioning

**Fig. 11:**
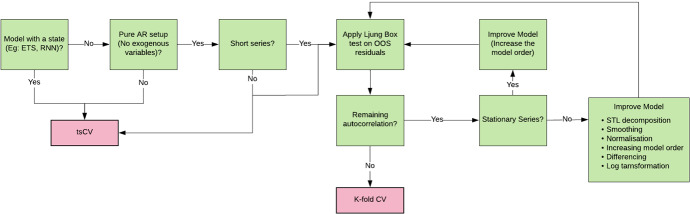
Flowchart for guidelines on data partitioning

It is important to identify which out of the above data partitioning strategies most closely estimates (without under/overestimation) the final error of a model for the test set under the given scenario (subject to different non-stationarities/serial correlations/amount of data of the given time series). The gist of the guidelines for data partitioning is visualized by the flow chart in Fig. 11. If the series are not short, tsCV is usually preferrable over k-fold CV, if there are no practical considerations such as that an implementation of an algorithm is used that is not primarily intended for time series forecasting, and that internally performs a certain type of cross-validation. If series are short, then k-fold CV should be used, accounting adequately for non-stationarities and autocorrelation in the residuals.

### Error measures for forecast evaluation

Once the predictions are obtained from models, the next requirement is to compute errors of the predictions to assess the model performance. Bias in predictions is a common issue and because of this, a model can be very accurate (forecasts being very close to actuals), but consistently produce more overestimations than underestimations, which may be concerning from a business perspective. Therefore, forecast bias is calculated with a sign, as opposed to absolute errors, so that it indicates the direction of the forecast errors, either positive or negative. For example, scale-dependent forecast bias can be assessed with the Mean Error (ME) as defined in Equation 4. Here, $y_t$ indicates the true value of the series, ${\hat{y}}_t$ the forecast and *n*, the number of all available errors. Other scale-free versions of bias can be defined by scaling with respect to appropriate scaling factors, such as actual values of the series.

4 $$\begin{aligned} \textit{ME} = \frac{1}{n}\sum _{t=1}^{n} \left( y_t - \hat{y_t}\right) \end{aligned}$$

Two other popular and simple error measures used in a usual regression context are MSE and MAE, which are both scale-dependent measures. Depending on the business context, it can be a valid objective to forecast more accurately the series that have higher scales, since they may be really the objects of interest. However, the problem with scale-dependent measures is that, as soon as the scale of the series is changed (for example converting from one currency to another), the value of the error measures change (Tashman 2000). On the other hand, for certain businesses, it is a requirement to compare errors across series. For example, if we say that MAE is 10 for a particular series, we have no idea whether it is a good or a bad accuracy. For a series with an average value of 1000, this amount of accuracy is presumably quite good, whereas for another series with an average value of 1, it is a very bad accuracy. For this reason, the measures need to be scaled to achieve scale-independent measures, and it has turned out to be next to impossible to develop a scaling procedure that works for any type of possible non-stationarity and non-normality in a time series. Hence, a wide variety of error measures have been proposed by researchers for this purpose over the years. Nevertheless, eventually we encounter a particular condition of the time series in the real world, that makes the proposed error measure fail (Svetunkov 2021). There are many options available for scaling such as per-step, per-series or per-dataset scaling. Scaling can also be done by dividing either by in-sample or OOS values of the time series. Apart from dividing by certain quantities, scaling can also be achieved through log transformation of errors and ranking based on errors as well. The key to selecting a particular error measure for forecast evaluation is that it is mathematically and practically robust under the given data.

Different point forecast evaluation measures are targeted towards optimizing for a specific statistic of the distribution. For example, measures with squared base errors such as MSE and RMSE optimize for the mean whereas others with absolute value base errors such as MAE and Mean Absolute Scaled Error (MASE) optimize for the median. Although the mean and median are the same for a symmetric distribution, that does not hold for skewed distributions as with intermittent series. There exist numerous controversies in the literature regarding this. Petropoulos (2022) suggest that it is not appropriate to evaluate the same forecasts using many different error measures, since each one optimizes for a different statistic of the distribution. Also according to Kolassa (2020), if different point forecast evaluation measures are considered, multiple point forecasts for each series and time point also need to be created. Kolassa (2020) further argues that, if the ultimate evaluation measure is, e.g., MAE which focuses on the median of the distribution, it does not make sense to optimize the models using an error measure like MSE (which accounts for the mean). It is more meaningful to consider MAE also during model training as well. However, these arguments hold only if it is not an application requirement for the same forecasts to perform generally well under all these measures. Koutsandreas et al. (2021) have empirically shown that, when the sample size is large, a wide variety of error measures agree on the most consistently dominating methods as the best methods for that scenario. They have also demonstrated that using two different error measures for optimizing and final evaluation has an insignificant impact on the final accuracy of the models. Bermúdez et al. (2006) have developed a fuzzy ETS model optimized via a multi-objective function combining three error measures MAPE, RMSE and MAE. Empirical results have demonstrated that using such a mix of error measures instead of just one for the loss function leads to overall better, robust and generalisable results even when the final evaluation is performed with just one of those measures. Fry and Lichtendahl (2020) also assess their same forecasts across numerous error measures in a business context. Evaluating the same forecasts with respect to many evaluation measures is a form of sanity checking to ensure that even under other measures (though not directly optimizing for them), the forecasts still perform well.

There are many different point forecast error measures available in the forecasting literature categorized based on 1) whether squared or absolute errors are used 2) techniques used to make them scale-free and 3) the operator such as mean, median used to summarize the errors (Koutsandreas et al. 2021). Also, there are different forms of base errors involved with each of the error measures. In the following base error definitions, $y_t$ indicates the true value of the series, ${\hat{y}}_t$ the forecast and *T*, the number of time steps in the training region of the time series.

- Scale-dependent base error
  5 $$\begin{aligned} e_t = y_t - {\hat{y}}_t \end{aligned}$$
- Percentage error
  6 $$\begin{aligned} p_t = \frac{100e_t}{y_t} \end{aligned}$$
- Percentage error (In-sample scaling) - Named as scaled Error (sE) in the work of Petropoulos and Kourentzes (2015).
  7 $$\begin{aligned} p^{\dagger }_t = \frac{e_t}{\frac{1}{T}\sum _{t=1}^{T}y_t} \end{aligned}$$
- Percentage absolute error (In-sample scaling) - Named as scaled Absolute Error (sAE) in the work of Petropoulos and Kourentzes (2015).
  8 $$\begin{aligned} p^{\ddagger }_t = \frac{|e_t|}{\frac{1}{T}\sum _{t=1}^{T}y_t} \end{aligned}$$
- Relative error - $e_t^b$ in Eq. (9) is the scale-dependent base error of the benchmark method.
  9 $$\begin{aligned} r_t = \frac{e_t}{e_t^b} \end{aligned}$$
- Scaled error (using MAE for the benchmark)
  10 $$\begin{aligned} q_t = \frac{e_t}{\frac{1}{T-1}\sum _{t=2}^{T}|y_t - y_{t-1}|} \end{aligned}$$
- Scaled error (using MSE for the benchmark)
  11 $$\begin{aligned} q_t^\dagger = \frac{e_t^2}{\frac{1}{T-1}\sum _{t=2}^{T}(y_t - y_{t-1})^2} \end{aligned}$$
- Logarithmic error - *ln* in Eq. (12) defines the natural logarithm.
  12 $$\begin{aligned} l_t = ln(y_t + 1) - ln({\hat{y}}_t + 1) \end{aligned}$$
  This is mathematically equivalent to the following.
  13 $$\begin{aligned} l_t = ln\left( \frac{y_t + 1}{{\hat{y}}_t + 1}\right) \end{aligned}$$
- Rate-based error (Kourentzes 2014)
  14 $$\begin{aligned} c_t = \hat{y_t} - \frac{1}{t}\sum _{i=1}^{t}y_i \end{aligned}$$

Table 8 contains the definitions of error measures proposed in the literature using the aforementioned base errors. In the definitions of Table 8, *n* indicates the number of all available base errors, *m* denotes the number of time series, *h* indicates the number of time steps in the forecast horizon and $h_i$, the horizon size for the $i^{th}$ series.

**Table 8:** Error measure definitions in the forecasting literature

Category	Error measure	Definition
Scale-Dependent Measures	Root Mean Squared Error (RMSE)	$\displaystyle \textit{RMSE} = \sqrt{\frac{1}{n}\sum _{t=1}^{n}(e_t^2)}$
	Root Median Squared Error (RMdSE)	$\displaystyle \textit{RMdSE} = \sqrt{ \textit{median}(e_t^2)}$
	Median Absolute Error (MdAE)	$\displaystyle \textit{MdAE} = \textit{median}(|e_t|)$
	Geometric Root Mean Squared Error (GRMSE, Syntetos and Boylan 2005)	$\displaystyle \textit{GRMSE} = \root 2n \of {\prod _{t=1}^{n}{e_t^2}}$
	Geometric Mean Absolute Error (GMAE)	$\displaystyle \textit{GMAE} = \root n \of {\prod _{t=1}^{n}{|e_t|}}$
Measures based on Percentage Errors	Mean Absolute Percentage Error (MAPE)	$\displaystyle \textit{MAPE} = \frac{1}{n}\sum _{t=1}^{n}(|p_t|)$
	Median Absolute Percentage Error (MdAPE)	$\displaystyle \textit{MdAPE} = \textit{median}(|p_t|)$
	Root Mean Square Percentage Error (RMSPE, Bojer and Meldgaard 2020)	$\displaystyle \textit{RMSPE} = \sqrt{\frac{1}{n}\sum _{t=1}^{n}(p_t^2)}$
	Root Median Square Percentage Error (RMdSPE)	$\displaystyle \textit{RMdSPE} = \sqrt{ \textit{median}(p_t^2)}$
	Symmetric Mean Absolute Percentage Error (sMAPE first proposed by Makridakis (1993))	$\displaystyle \textit{sMAPE} = \frac{1}{n}\sum _{t=1}^{n}(\frac{200|e_t|}{|y_t| + |{\hat{y}}_t|})$
	Symmetric Median Absolute Percentage Error (sMdAPE)	$\displaystyle \textit{sMdAPE} = \textit{median}(\frac{200|e_t|}{|y_t| + |{\hat{y}}_t|})$
	Modified Symmetric Mean Absolute Percentage Error (msMAPE, Suilin 2017)	$\displaystyle \textit{msMAPE} = \frac{1}{n}\sum _{t=1}^{n}\frac{200|e_t|}{ \textit{max}(|y_t| + |{\hat{y}}_t| + \epsilon , 0.5 + \epsilon )}$, where $\epsilon = 0.1$ by default
	Mean Arctangent Absolute Percentage Error (MAAPE, Kim and Kim 2016)	$\displaystyle \textit{MAAPE} = \frac{1}{n}\sum _{t=1}^{n} \textit{arctan}(|\frac{e_t}{y_t}|)$
	Weighted Absolute Percentage Error (WAPE)	$\displaystyle \textit{WAPE} = \frac{\sum _{t=T+1}^{T+h}|e_t|}{\sum _{t=T+1}^{T+h}|y_t|}$
	Symmetric Weighted Absolute Percentage Error (sWAPE)	$\displaystyle \textit{sWAPE} = \frac{\sum _{t=T+1}^{T+h}|e_t|}{\sum _{t=T+1}^{T+h}|y_t| + |{\hat{y}}_t|}$
	Weighted Root Mean Squared Percentage Error (WRMSPE)	$\displaystyle \textit{WRMSPE} = \sqrt{\frac{\frac{1}{h}\sum _{t=T+1}^{T+h}e^2_t}{\frac{1}{h}\sum _{t=T+1}^{T+h}|y_t|}}$
	Relative Total Absolute Error (RTAE)	$\displaystyle \textit{RTAE} = \frac{\frac{1}{h}\sum _{t=T+1}^{T+h}|e_t|}{max(C, \frac{1}{h}\sum _{t=T+1}^{T+h}|y_t|)}$, where *C* refers to a regularisation threshold
	Scaled Mean Error (sME, Petropoulos and Kourentzes 2015)	$\displaystyle \textit{sME} = \frac{1}{n}\sum _{t=1}^{n}({p^{\dagger }_t})$
	Scaled Mean Squared Error (sMSE, Petropoulos and Kourentzes 2015)	$\displaystyle \textit{sMSE} = \frac{1}{n}\sum _{t=1}^{n}({p^{\dagger }_t}^2)$
	Scaled Mean Absolute Error (sMAE, Petropoulos and Kourentzes 2015)	$\displaystyle \textit{sMAE} = \frac{1}{n}\sum _{t=1}^{n}({p^{\ddagger }_t})$
	Normalized Deviation (ND, Salinas et al. 2020) - The scale in the denominator is computed globally using many series.	$\displaystyle \textit{ND} = \frac{\sum _{t=1}^{n}|e_{t}|}{\sum _{t=1}^{n}|y_{t}|}$
	Normalized Root Mean Squared Error (NRMSE, Salinas et al. 2020) - The scale in the denominator is computed globally using many series.	$\displaystyle \textit{NRMSE} = \frac{\sqrt{\frac{1}{n}\sum _{t=1}^{n}(e_{t}^2)}}{\frac{1}{n}\sum _{t=1}^{n}(|y_{t}|)}$
Measures based on Relative Errors	Mean Relative Absolute Error (MRAE)	$\displaystyle \textit{MRAE} = \frac{1}{n}\sum _{t=1}^{n}(|r_t|)$
	Median Relative Absolute Error (MdRAE)	$\displaystyle \textit{MdRAE} = \textit{median}(|r_t|)$
	Root Mean Relative Squared Errors (RMRSE)	$\displaystyle \textit{RMRSE} = \sqrt{\frac{1}{n}\sum _{t=1}^{n}(r^2_t)}$
	Geometric Mean Relative Absoluate Error (GMRAE)	$\displaystyle \textit{GMRAE} = \root n \of {\prod _{t=1}^{n}{|r_t|}}$
	Relative Geometric Root Mean Squared Error (RGRMSE)	$\displaystyle \textit{RGRMSE} = \root 2n \of {\prod _{t=1}^{n}{r_t^2}}$
Relative Measures	Relative Mean Absolute Error (RelMAE)	$\displaystyle \textit{RelMAE} =\frac{ \textit{MAE}}{ \textit{MAE}_b}$, where $\textit{MAE}_b$ is the $\textit{MAE}$ of the benchmark method
	Relative Mean Squared Error (RelMSE)	$\displaystyle \textit{RelMSE} = \frac{ \textit{MSE}}{ \textit{MSE}_b}$, where $\textit{MSE}_b$ is the $\textit{MSE}$ of the benchmark method
	Relative Root Mean Squared Error (RelRMSE)	$\displaystyle \textit{RelRMSE} = \sqrt{\frac{ \textit{MSE}}{ \textit{MSE}_b}}$, where $\textit{MSE}_b$ is the $\textit{MSE}$ of the benchmark method
	Root Relative Squared Error (RSE, Lai et al. 2018)	$\displaystyle \textit{RSE} = \frac{\sqrt{\sum _{t=1}^{n}e_{t}^2}}{\sqrt{\sum _{t=1}^{n}(y_{t} - {\bar{y}})^2}}$
	Average Relative Mean Absolute Error (AvgRelMAE, Davydenko and Fildes 2013)	$\displaystyle \textit{AvgRelMAE} = \left( \prod _{i=1}^{m}{\left( \frac{ \textit{MAE}^i}{ \textit{MAE}_b^i}\right) ^{h_i}}\right) ^{\frac{1}{\sum _{i=1}^{m}h_i}}$, where $\textit{MAE}_b^i$ is the $\textit{MAE}$ of the benchmark method for the $i^{th}$ series
Measures based on Scaled Errors (Hyndman and Koehler 2006)	Mean Absolute Scaled Error (MASE)	$\displaystyle \textit{MASE} = \frac{1}{n}\sum _{t=1}^{n} q_t$
	Median Absolute Scaled Error (MdASE)	$\displaystyle \textit{MdASE} = \textit{median}(q_t)$
	Root Mean Squared Scaled Error (RMSSE, Makridakis et al. 2022)	$\displaystyle \textit{RMSSE} = \sqrt{\frac{1}{n}\sum _{t=1}^{n}q_t^\dagger }$
Measures based on Ranks/Counting	Percentage Better (PB Score, Hyndman and Koehler 2006) - Counts how many times (across series and time steps) a given method is better than the benchmark and reports it as a percentage.	$\displaystyle \textit{PB(MAE)} = 100\ \textit{mean}(I\{ \textit{MAE}< \textit{MAE}_b\})$, where $\textit{MAE}_b$ is the $\textit{MAE}$ of the benchmark method.
	Percentage of Critical Event for Margin X - Wong (2019) proposed this to measure the percentage of forecasts where the value of error is higher than a margin.	$\displaystyle 100\ \textit{mean}(I\{E>X\})$, where *E* is the error and *X* is the margin
Measures based on Transformation	Root Mean Squared Logarithmic Error (RMSLE, Bojer and Meldgaard 2020)	$\displaystyle \textit{RMSLE} = \sqrt{\frac{1}{n}\sum _{t=1}^{n}l_t}$
	Normalized Weighted Root Mean Squared Logarithmic Error (NWRMSLE, Bojer and Meldgaard 2020)	$\displaystyle \textit{NWRMSLE} = \sqrt{\frac{\sum _{t=1}^{n}w_tl_t^2}{\sum _{t=1}^{n}w_t}}$, where $w_t$ is a weight assigned to the error at time step *t*
Rate-based Measures (Kourentzes 2014)	Mean Squared Rate (MSR)	$\displaystyle \textit{MSR} = \sum _{t=1}^{n}c_t^2$
	Mean Absolute Rate (MAR)	$\displaystyle \textit{MAR} = \sum _{t=1}^{n} |c_t|$
Other Error Measures	Weighted Mean Absolute Error (WMAE, Bojer and Meldgaard 2020)	$\displaystyle \textit{WMAE} = \frac{\sum _{t=1}^{n}w_t|e_t|}{\sum _{t=1}^{n}w_t}$, where $w_t$ is a weight assigned to the error at time step *t*
	Empirical Correlation Coefficient (CORR, Lai et al. 2018)	$\displaystyle \textit{CORR} = \frac{1}{m}\sum _{i=1}^{m}(\frac{\sum _{t=T+1}^{T + h}(y_{it} - \bar{y_i})({\hat{y}}_{it} - \bar{\hat{y_i}})}{\sqrt{\sum _{t = T+1}^{T+h}(y_{it} - \bar{y_i})^2({\hat{y}}_{it} - \bar{\hat{y_i}})^2}})$, where $\bar{y_i}$ is the mean of series *i* and $\bar{\hat{y_i}}$ is the mean of the predictions for series *i*

Depending on each of the characteristics of time series as also stated in Sect. 2, different error measures defined in Table 8 are preferable or should be avoided in each case. Table 9 summarises this information and can be used to choose error measures under given characteristics of the data. In Table 9, the scaling column indicates the type of scaling associated with each error measure mentioned in the previous column. This includes no scaling, scaling based on actual values, scaling based on benchmark errors as well as the categorisation such as per-step, per-series and all-series (per-dataset) scaling. The $\dagger$ sign in Table 9 indicates that the respective error measures need to be used with caution under the given circumstances.

In almost any scenario, when applying error measures that scale based on errors from a benchmark method, the relative competence of the benchmark method in the intended forecast horizon needs to be taken into account, since otherwise benchmark errors can unnecessarily drive the overall error measure values higher or lower. With series having seasonality, percentage based measures may underestimate the errors at peaks heavily, due to dividing by large actual values (Wong 2019; Kunst 2016) or overstate the errors at troughs. This can be overcome by scaling based on aggregated values (per series, all-series). On series having trends or structural breaks with level shifts, scale-free measures which compute their scale by aggregating the values (actual values or benchmark errors) at several time steps, tend to face problems. This is as explained by Chen et al. (2017), that the error values at each time step need to comply with the scale of the series at each point. A scale computed by aggregating over several time steps which include such level shifts may not always be a good estimator to represent the scaling factors for all the time steps of such a series. Also on series with exponential trends, log transformation based error measures greatly reduce the impact of errors from models. Unit roots are very similar to trends except that measures which compute a per-step scaling may not capture peak points on such series similar to seasonal series.

Similarly, on series having heteroscedasticity too, due to potential peaks and troughs in the series which may have very high and low variances, measures such as MAPE and RMSPE may have problems with capturing those points correctly. Apart from that, log transformation based errors can reduce the impact from heteroscedasticity as well. Especially on series having structural breaks, with measures which scale based on benchmark errors, when those errors are computed in-sample, they may not be representative of the errors that happen OOS when the structural breaks are either in the forecast horizon or the forecast origin. On intermittent series, measures that optimize for the median are problematic since they consider constant zeros as the best prediction. Measures with per-step scaling based on actual values can also be problematic on intermittent series due to dividing by zero. This can be addressed by using per-series scaling, but can again have issues if all time steps have zero values. With measures that scale based on benchmark errors on intermittent series, it can be problematic when benchmark errors have prefect predictions (zero errors), for example with the naïve method giving exact zeros on zero actual values. With respect to outliers, some applications may be interested in capturing them whereas others may want to be robust against them. To be robust against outliers, geometric mean or median can be used as the summary operator instead of the mean. Absolute base errors need to be used instead of squared base errors to be robust against outliers. Measures which scale based on per-step or per-series quantities may be heavily affected by outliers. Similarly, with measures that scale based on benchmark errors, if the forecast of the benchmark in the horizon is heavily affected by the outliers in the training region of the series, it can be problematic.

**Fig. 12:**
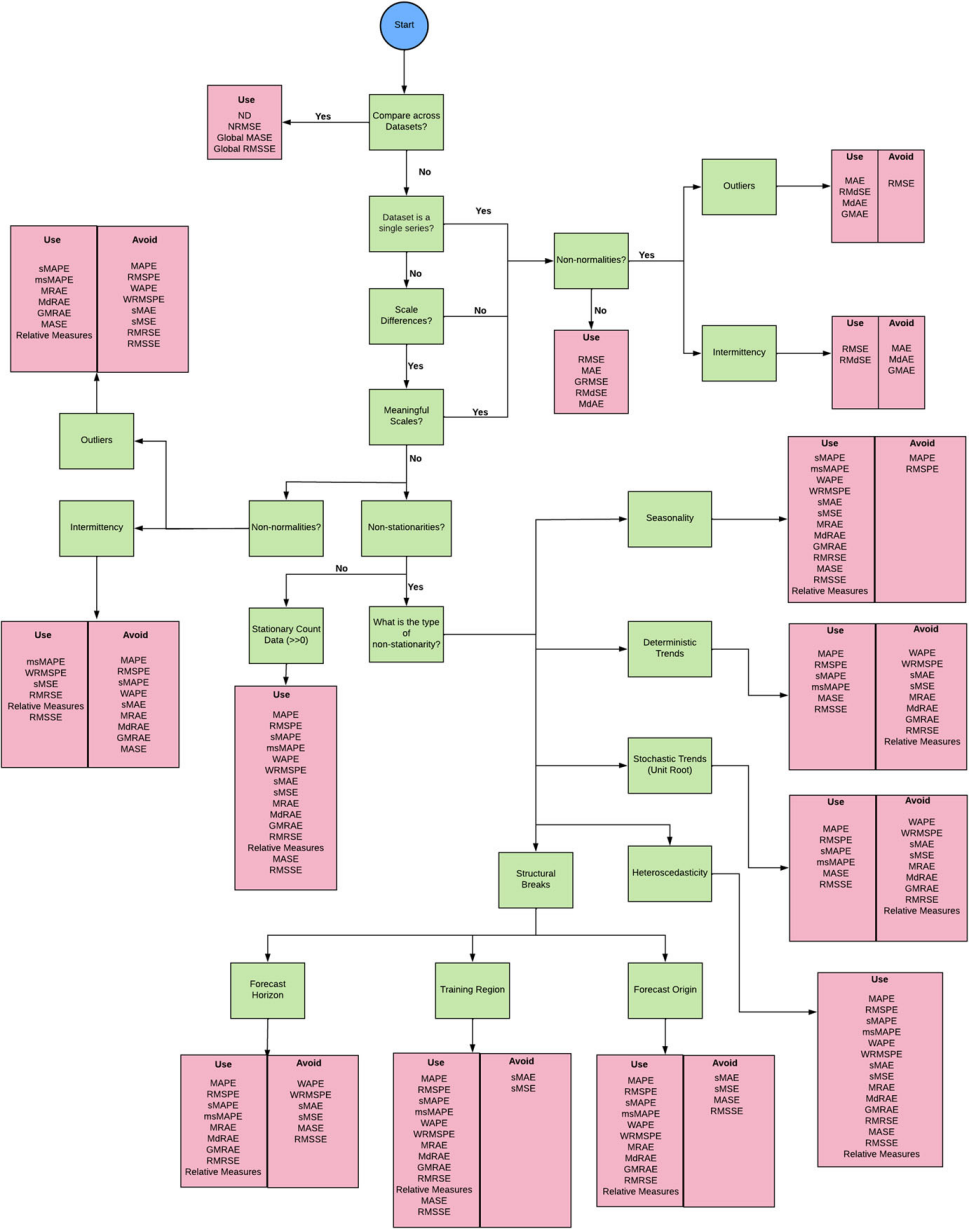
Flow chart for forecast error measure selection

The flow chart in Fig. 12 provides further support for forecast evaluation measure selection based on user requirements and other characteristics in the data. In Fig. 12, the error measures selected to be used with outlier time series are in the context of being robust against outliers, not capturing them.

**Table 9:** Checklist for selecting error measures for final forecast evaluation based on different time series characteristics

Stationary Count ) Data(>> 0	Seasonality	Trend (Linear/Exp.)	Unit Roots	Heteroscedasticity	Structural Breaks (With Scale Differences)			Intermittence	Outliers	Error Measures	Scaling	
					Forecast Horizon	Training Region	Forecast Origin					
✓	✓	✓	✓	✓	✓	✓	✓	✓	✗	RMSE	None	
✓	✓	✓	✓	✓	✓	✓	✓	✗	✓	MAE		
✓	✗	✓	✓^†^	✓^†^	✓	✓	✓	✗	✗	MAPE	OOS Per Step	Actual Values
✓	✗	✓	✓^†^	✓^†^	✓	✓	✓	✗	✗	RMSPE		
✓	✓	✓	✓	✓	✓	✓	✓	✗	✓	sMAPE		
✓	✓	✓	✓	✓	✓	✓	✓	✓	✓	msMAPE		
✓	✓	✗	✗	✓	✗	✓	✓	✗	✗	WAPE	OOS Per Series	
✓	✓	✗	✗	✓	✗	✓	✓	✓^†^	✗	WRMSPE		
✓	✓	✗	✗	✓	✗	✗	✗	✗	✗	sMAE	In-Sample Per Series	
✓	✓	✗	✗	✓	✗	✗	✗	✓^†^	✗	sMSE		
✓	✓	✓	✓	✓	✓	✓	✓	✗	✓	ND	OOS All Series	
✓	✓	✓	✓	✓	✓	✓	✓	✓	✗	NRMSE		
✓^†^	✓^†^	✗	✗	✓	✓	✓^†^	✓	✗	✓^†^	MRAE	OOS Per Step	Benchmark Errors
✓^†^	✓^†^	✗	✗	✓	✓	✓^†^	✓	✗	✓^†^	MdRAE		
✓^†^	✓^†^	✗	✗	✓	✓	✓^†^	✓	✗	✓^†^	GMRAE		
✓^†^	✓^†^	✗	✗	✓	✓	✓^†^	✓	✓^†^	✗	RMRSE		
✓^†^	✓^†^	✗	✗	✓	✓	✓^†^	✓	✓^†^	✓^†^	Relative Measures	OOS Per Series	
✓^†^	✓^†^	✓	✓	✓	✗	✓^†^	✗	✗	✓	MASE	In-Sample PerSeries	
✓^†^	✓^†^	✓	✓	✓	✗	✓^†^	✗	✓^†^	✗	RMSSE		
✓^†^	✓^†^	✓	✓	✓	✓	✓	✓	✓	✓		In-Sample All Series	
✓	✓	✓^†^	✓	✓^†^	✓	✓	✓	✓	✓	Measures with Transformations	None	

### Statistical tests for significance

While forecast evaluation measures are critical to see the relative performance of the methods and select the best ones from their rankings, they do not give information regarding the statistical significance of the differences between these methods; i.e. whether better performance of the best method is just by chance on this sample of the series or whether it is likely to dominate all the methods significantly in other samples of the data. The selected best method could be the only one to use, or there could be other methods that are not significantly different from the best that can be used interchangeably due to their other preferable properties such as simplicity, computational efficiency etc.

There are many ways of performing statistical significance tests reported in the literature. The Diebold-Mariano test (Diebold and Mariano 2002) and the Wilcoxon rank-sum test (Mann and Whitney 1947) are both designed for comparing only between two competing forecasts, not necessarily methods or models. However, the Diebold-Mariano test is designed specifically for time series and parametric, meaning that it has the assumption of normality of the data whereas the Wilcoxon test is a generic non-parametric test based on the ranks of the methods. Due to considering ranks of methods for each series separately, the error measures used do not necessarily have to be scale-free. The Giacomini-White test (Giacomini and White 2006) again is based on the comparison of two forecasts, with the potential to assess the conditional predictive ability (CPA), a concept that refers to conditioning the choice of a potential future state of the economy, an important concept for macro economic forecasting of a small number of series. A continuation in this line of research is work by Li et al. (2022b) that focuses on conditional superior predictive ability, in regards to a benchmark method and time series with general serial dependence. It should be noted that many of the mentioned comparison tests are per-se designed for comparing two forecasts, and a multiple testing of more than two requires a correction for multiple hypothesis testing, such as, e.g., a Bonferroni correction.

There are other techniques developed to perform comparison within a group of methods (more than 2) as well. Means of error distributions from different methods can be used to compare the mean performance of the methods. The F-test and the t-test are statistical tests in this respect. They both have parametric assumptions for the means of the error distributions, that they need to follow a normal distribution. Although, according to the Central Limit Theorem, this could hold for measures such as MSE, MAE etc., for a sufficiently large random sample (of size $n\ge 30$), it does not hold for e.g., RMSE, since the root of a normally distributed variable is following a chi-square distribution, which is close to normality but not equivalent. On the other hand, the Friedman test (Friedman 1937, 1939, 1940) is a non-parametric statistical test that can be used to detect significance between multiple competing methods, using the ranks of the methods according to mean errors.

**Fig. 13:**
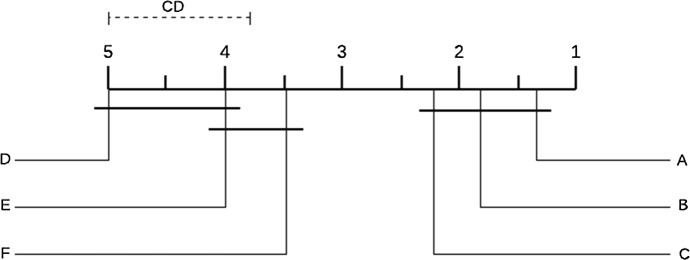
An example of a CD diagram to visualize the significance of the differences between a number of competing methods. The best three methods A, B and C are not significantly different from each other. On the other hand, methods D, E and F are significantly worse than those three methods. The amount of data has not been enough to check whether method E is significantly better than method D or worse than method F

The Friedman test is usually followed by a post-hoc test, when the null hypothesis which states that “there are no significant differences between the methods”, is rejected. There are different types of post-hoc tests, for example, the Hochberg procedure (Hochberg 1988), the Holm process (Holm 1979), the Bonferroni-Dunn procedure (Dunn 1961), the Nemenyi method (Nemenyi 1963), the Multiple Comparisons with the Best (MCB) method (practically equivalent to the Nemenyi method) or the Multiple Comparisons with the Mean (ANOM) method (Halperin et al. 1955), and others. In general, the ANOM test holds less value in practice since it is more useful to find which methods are not significantly different from the best, than from some averagely performing method overall. The Nemenyi method works by defining confidence bounds, in terms of a Critical Distance (CD) around the mean ranks of the methods to identify which methods have overlapping confidence bounds and which do not. As Demšar (2006) suggests, if all the comparisons are to be performed against one control method as opposed to each method against each other, procedures such as Bonferroni-Dunn and Hochberg’s are better over the Nemenyi test. Once, the quantitative results for the significance of the differences are obtained using any of the aforementioned methods, they can be visualized using CD diagrams (Demšar 2006). In general, in these diagrams, a horizontal axis reports the average ranks of all the methods. Groups of methods that are not significantly different from each other are connected using black bars. This is illustrated in Fig. 13, an example CD diagram.

**Fig. 14:**
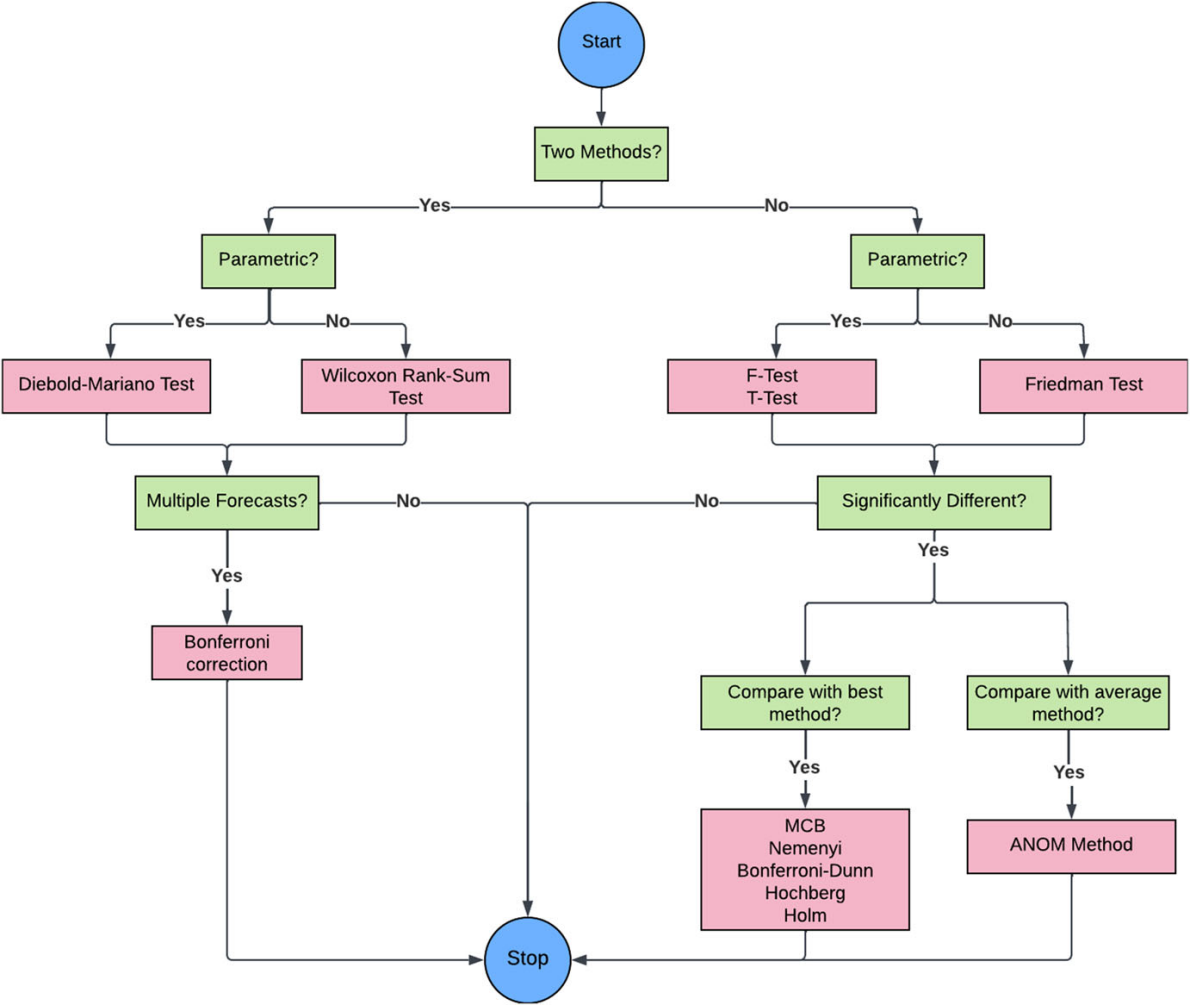
Flow chart for statistical tests selection to measure significance of model differences

When performing significance testing, the amount of data included heavily impacts the results of the significance tests. For example, with a very high number of series, the CD is usually very low, producing significant results for even small differences between models. This means that the results are more reliable, that even the slightest differences between models encountered for such a large amount of data are statistically highly significant. On the other hand, it also depends on the number and the relative performance of the set of models included in the comparison. For example, having more and more poorly performing methods in the group may tend towards making the CD larger, thus making other intermediate methods have no significant difference from the best. The flow chart in Fig. 14 summarises the decision making process in selecting a statistical test to measure significance of differences between models.

## Conclusions

Model evaluation, just as in any other domain, is a crucial step in forecasting. In other major fields such as regression, classification, there exist established techniques that are the standard best practices. On the contrary, in the domain of forecasting, evaluation remains a much more complex task. The general trend in the literature has been to propose new methodologies to address pitfalls associated with the previously introduced. Nevertheless, for example with the forecast evaluation measures, to the best of our knowledge, all the introduced measures thus far can break under given certain characteristics/non-stationarities of the time series. General ML practitioners and Data Scientists new to the field of forecasting are often not aware of these issues. Consequently, as we demonstrate through our work, forecast evaluation practices used by many works even published at top-tier venues in the ML domain can be flawed. All of this is a consequence of the lack of established best practices and guidelines for the different steps of the forecast evaluation process. Therefore, to support the ML community in this aspect, we provide a compilation of common pitfalls and best practice guidelines related to forecast evaluation. The key set of guidelines that we develop are as follows.

- To claim the competitiveness of the proposed methods, they need to be benchmarked on sufficiently large amounts of datasets.
- It is always important to compare models against the right and the simplest benchmarks such as the naïve and the seasonal naïve.
- Using forecast plots can be misleading; making decisions purely based on the visual appeal on forecast plots is not advisable.
- Data leakage needs to be avoided explicitly in rolling origin evaluation and other data pre-processing tasks such as smoothing, decomposition and normalisation of the series.
- If enough data are available, tsCV is the procedure of choice. Also, for models with a continuous state such as RNNs and ETS where the temporal order of the data is important, tsCV may be the only applicable validation strategy. k-fold CV is a valid and a data efficient strategy of data partitioning for forecast model validation with pure AR based setups, when the models do not underfit the data (which can be detected with a test for serial correlation in the residuals, such as the Ljung-Box test). As such, we advise this procedure especially for short series where tsCV leads to test sets that are too small. However, if the models underfit, it is advisable to improve the models first before using any CV technique.
- There is no single globally accepted evaluation measure for all scenarios. It depends on the characteristics of the data as summarized in Table 9.
- When using statistical testing for significance of the differences between models, balancing the diversity of the compared models against the number of data points is important to avoid spurious statistical similarity/difference between models.

While the literature on evaluation measures is quite extensive, the exact errors (squared/ absolute), summarisation operators (mean/median/geometric mean), type of scaling to use (global/per-series/per-step/, in-sample/OOS, relative/percentage) differ based on the user expectations, business utility and the characteristics of the underlying time series. Due to the lack of proper knowledge in forecast evaluation, ML research in the literature thus far has often either struggled to demonstrate the competitiveness of its models or arrived at spurious conclusions. It is our objective that this effort encourages better and correct forecast evaluation practices within the ML community. As a potential avenue for further work especially with respect to evaluation measures, it would be useful to design combination based evaluation measures for forecasting, similar to the Huber loss for model training, which is a combination of the MAE and the RMSE. These types of measures can be quite robust, combining the strengths of both measures while minimising the potential disadvantages associated with the individual measures.

## Footnotes

1 Available at https://github.com/HansikaPH/Forecast_Evaluation_Pitfalls.

2 https://archive.ics.uci.edu/ml/datasets/ElectricityLoadDiagrams20112014

3 One could argue that not always the true data generating process (DGP) is the best forecasting model, but this usually happens for complex DGPs where not enough data is available to estimate their parameters correctly and so simpler models perform better for forecasting. However, an AR(1) is already very simple so that compared to a considerably more complex neural network this consideration seems not relevant here.

## References

[CR1] Armstrong J, Armstrong JS (2001). Evaluating forecasting methods. Principles of forecasting: a handbook for researchers and practitioners.

[CR2] Armstrong JS, Grohman MC (1972). A comparative study of methods for long-range market forecasting. Manag Sci.

[CR3] Arnott R, Harvey C R, Markowitz H (2019) A backtesting protocol in the era of machine learning. J Financ Data Sci

[CR4] Bagnall A, Lines J, Bostrom A, Large J, Keogh E (2016). The great time series classification bake off: a review and experimental evaluation of recent algorithmic advances. Data Min Knowl Disc.

[CR5] Balestriero R, Pesenti J, LeCun Y (2021) Learning in high dimension always amounts to extrapolation. arXiv preprint arXiv:2110.09485

[CR6] Bell F, Smyl S, (2018) Forecasting at uber: an introduction. https://eng.uber.com/forecasting-introduction/

[CR7] Berger D, Chaboud A, Hjalmarsson E (2009). What drives volatility persistence in the foreign exchange market?. J Financ Econ.

[CR8] Bergmeir C, Hyndman RJ, Koo B (2018). A note on the validity of cross-validation for evaluating autoregressive time series prediction. Comput Stat Data Anal.

[CR9] Bermúdez JD, Segura JV, Vercher E (2006). A decision support system methodology for forecasting of time series based on soft computing. Comput Stat Data Anal.

[CR10] Bojer C S, Meldgaard J P (2020) Kaggle forecasting competitions: an overlooked learning opportunity. Int J Forecast

[CR11] Brownlee J (2020) Data preparation for machine learning: data cleaning, feature selection, and data transforms in Python. Mach Learn Mastery

[CR12] Cerqueira V, Torgo L, Mozetič I (2020). Evaluating time series forecasting models: an empirical study on performance estimation methods. Mach Learn.

[CR13] Challu C, Olivares K. G, Oreshkin B N, Garza, F, Mergenthaler-Canseco M, Dubrawski A (2022) N-hits: neural hierarchical interpolation for time series forecasting. arXiv:2201.12886

[CR14] Chen C, Twycross J, Garibaldi JM (2017). A new accuracy measure based on bounded relative error for time series forecasting. PLoS ONE.

[CR15] Cox D, Miller H (1965) The Theory of Stochastic Processes

[CR16] Cui Y, Xie J, Zheng K (2021) Historical inertia: a neglected but powerful baseline for long sequence time-series forecasting. In: Proceedings of the 30th ACM International Conference on Information & Knowledge Management. CIKM ’21. Association for Computing Machinery, New York, NY, USA, pp 2965-2969

[CR17] Davydenko A, Fildes R (2013). Measuring forecasting accuracy: The case of judgmental adjustments to SKU-level demand forecasts. Int J Forecast.

[CR18] Demšar J (2006). Statistical comparisons of classifiers over multiple data sets. J Mach Learn Res.

[CR19] Diebold FX, Mariano RS (2002). Comparing predictive accuracy. J Bus Econ Stat.

[CR20] Ditzler G, Roveri M, Alippi C, Polikar R (2015). Learning in nonstationary environments: a survey. IEEE Comput Intell Mag.

[CR21] Dunn OJ (1961). Multiple comparisons among means. J Am Stat Assoc.

[CR22] Du D, Su B, Wei Z (2022) Preformer: predictive transformer with multi-scale segment-wise correlations for long-term time series forecasting. arXiv:2202.11356

[CR23] Du Y, Wang J, Feng W, Pan S, Qin T, Xu R, Wang C (2021) Adarnn: adaptive learning and forecasting of time series. In: Proceedings of the 30th ACM International Conference on Information & Knowledge Management. CIKM ’21. Association for Computing Machinery, New York, NY, USA, pp 402-411

[CR24] Engle R F (2003) Risk and volatility: econometric models and financial practice. Nobel Lect. https://www.nobelprize.org/uploads/2018/06/engle-lecture.pdf

[CR25] Fama EF (1970). Efficient capital markets: a review of theory and empirical work. J Financ.

[CR26] Fawaz HI, Forestier G, Weber J, Idoumghar L, Muller P-A (2019). Deep learning for time series classification: a review. Data Min Knowl Discov.

[CR27] Friedman M (1937). The use of ranks to avoid the assumption of normality implicit in the analysis of variance. J Am Stat Assoc.

[CR28] Friedman M (1939). A correction: the use of ranks to avoid the assumption of normality implicit in the analysis of variance. J Am Stat Assoc.

[CR29] Friedman M (1940). A comparison of alternative tests of significance for the problem of $$m$$ rankings. Ann Math Stat.

[CR30] Fry C, Lichtendahl C (2020) Google practitioner session. In: 40th International Symposium on Forecasting. https://www.youtube.com/watch?v=FoUX-muLlB4 &t=3007s

[CR31] Gama J, Sebastiao R, Rodrigues PP (2013). On evaluating stream learning algorithms. Mach Learn.

[CR32] Gama J. a, Žliobaitundefined I, Bifet A, Pechenizkiy M, Bouchachia A (2014) A survey on concept drift adaptation. ACM Comput Surv 46 (4)

[CR33] Ghomeshi H, Gaber MM, Kovalchuk Y (2019). EACD: evolutionary adaptation to concept drifts in data streams. Data Min Knowl Disc.

[CR34] Giacomini R, White H (2006). Tests of conditional predictive ability. Econometrica.

[CR35] Godahewa R, Bandara K, Webb GI, Smyl S, Bergmeir C (2021). Ensembles of localised models for time series forecasting. Knowl Based Syst.

[CR36] Godfrey LB, Gashler MS (2018). Neural decomposition of time-series data for effective generalization. IEEE Trans Neural Netw Learn Syst.

[CR37] Gujarati DN (2021). Essentials of econometrics.

[CR38] Guo Y, Zhang S, Yang J, Yu G, Wang Y (2022). Dual memory scale network for multi-step time series forecasting in thermal environment of aquaculture facility: a case study of recirculating aquaculture water temperature. Expert Syst Appl.

[CR39] Halperin M, Greenhouse SW, Cornfield J, Zalokar J (1955). Tables of percentage points for the studentized maximum absolute deviate in normal samples. J Am Stat Assoc.

[CR40] Hämäläinen W, Webb G I, (2019) A tutorial on statistically sound pattern discovery. Data Min Knowl Discov 33 (2): 325–377

[CR41] Hannun A, Guo C, van der Maaten L (2021) Measuring data leakage in machine-learning models with fisher information. In: de Campos, C, Maathuis, M H (eds) Proceedings of the Thirty-Seventh Conference on Uncertainty in Artificial Intelligence. vol 161, pp 760–770

[CR42] Hastie T, Tibshirani R, Friedman J (2009). The elements of statistical learning: data mining, inference, and prediction.

[CR43] Hewamalage H, Bergmeir C, Bandara K (2021). Recurrent neural networks for time series forecasting: current status and future directions. Int J Forecast.

[CR44] Hochberg Y (1988). A sharper bonferroni procedure for multiple tests of significance. Biometrika.

[CR45] Holm S (1979). A simple sequentially rejective multiple test procedure. Scand J Stat.

[CR46] Hyndman R J, Athanasopoulos G (2018) Forecasting: principles and Practice, 2nd edn. OTexts. https://otexts.com/fpp2/

[CR47] Hyndman RJ, Koehler AB (2006). Another look at measures of forecast accuracy. Int J Forecast.

[CR48] Hyndman R, Kang Y, Talagala T, Wang E, Yang Y (2019) tsfeatures: time series feature extraction. R package version 1.0.0. https://pkg.robjhyndman.com/tsfeatures/

[CR49] Ikonomovska E, Gama J, Džeroski S (2010). Learning model trees from evolving data streams. Data Min Knowl Discov.

[CR50] Kaufman S, Rosset S, Perlich C, Stitelman O (2012). Leakage in data mining: Formulation, detection, and avoidance. ACM Trans Knowl Discov Data.

[CR51] Kim S, Kim H (2016). A new metric of absolute percentage error for intermittent demand forecasts. Int J Forecast.

[CR52] Kolassa S (2020) Why the best point forecast depends on the error or accuracy measure. Int J Forecast 36(1):208–211

[CR53] Kourentzes N (2014). On intermittent demand model optimisation and selection. Int J Prod Econ.

[CR54] Koutsandreas D, Spiliotis E, Petropoulos F, Assimakopoulos V (2021) Aasures. J Oper Res Soc, 1–18

[CR55] Kunst R (2016) Visualization of distance measures implied by forecast evaluation criteria. In: International Symposium on Forecasting 2016. https://forecasters.org/wp-content/uploads/gravity_forms/7-621289a708af3e7af65a7cd487aee6eb/2016/07/Kunst_Robert_ISF2016.pdf

[CR56] Kuranga C, Pillay N (2022). A comparative study of nonlinear regression and autoregressive techniques in hybrid with particle swarm optimization for time-series forecasting. Expert Syst Appl.

[CR57] Lai G, Chang W.-C, Yang Y, Liu H (2018) Modeling long- and short-term temporal patterns with deep neural networks. In: The 41st International ACM SIGIR Conference on Research & Development in Information Retrieval. SIGIR ’18. Association for Computing Machinery, New York, NY, USA, pp 95-104

[CR58] Li J, Liao Z, Quaedvlieg R (2022). Conditional superior predictive ability. Rev Econ Stud.

[CR59] Li B, Du S, Li T, Hu J, Jia Z (2022a) Draformer: differentially reconstructed attention transformer for time-series forecasting. arXiv:2206.05495

[CR60] Lin G, Lin A, Cao J (2021). Multidimensional knn algorithm based on eemd and complexity measures in financial time series forecasting. Expert Syst Appl.

[CR61] Liu S, Ji H, Wang MC (2020). Nonpooling convolutional neural network forecasting for seasonal time series with trends. IEEE Trans Neural Netw Learn Syst.

[CR62] Liu Q, Long L, Peng H, Wang J, Yang Q, Song X, Riscos-Núñez A, Pérez-Jiménez M J (2021) Gated spiking neural p systems for time series forecasting. IEEE Trans Neural Netw Learn Syst, 1–10 10.1109/TNNLS.2021.3134792 34936560

[CR63] Ljung GM, Box GEP (1978). On a measure of lack of fit in time series models. Biometrika.

[CR64] Lubba CH, Sethi SS, Knaute P, Schultz SR, Fulcher BD, Jones NS (2019). catch22: CAnonical time-series CHaracteristics. Data Min Knowl Disc.

[CR65] Makridakis S (1993). Accuracy measures: theoretical and practical concerns. Int J Forecast.

[CR66] Makridakis S, Hibon M (2000). The m3-competition: results, conclusions and implications. Int J Forecast.

[CR67] Makridakis S, Spiliotis E, Assimakopoulos V (2020). The M4 Competition: 100,000 time series and 61 forecasting methods. Int J Forecast.

[CR68] Makridakis S, Spiliotis E, Assimakopoulos V (2022). M5 accuracy competition: results, findings, and conclusions. Int J Forecast.

[CR69] Mann HB, Whitney DR (1947). On a test of whether one of two random variables is stochastically larger than the other. Ann Math Stat.

[CR70] Moon H, Lee H, Song B (2022). Mixed pooling of seasonality for time series forecasting: an application to pallet transport data. Expert Syst Appl.

[CR71] Nemenyi P (1963) Distribution-free multiple comparisons. In: Ph.D. thesis, Princeton University

[CR72] Petropoulos F (2022). Forecasting: theory and practice. Int J Forecast.

[CR73] Petropoulos F, Kourentzes N (2015). Forecast combinations for intermittent demand. J Oper Res Soc.

[CR74] Ran P, Dong K, Liu X, Wang J (2023). Short-term load forecasting based on ceemdan and transformer. Electric Power Sys Res.

[CR75] Rossi B (2013). Exchange rate predictability. J Econ Lit.

[CR76] Salinas D, Flunkert V, Gasthaus J, Januschowski T (2020). Deepar: probabilistic forecasting with autoregressive recurrent networks. Int J Forecast.

[CR77] Salles R, Belloze K, Porto F, Gonzalez PH, Ogasawara E (2019). Nonstationary time series transformation methods: an experimental review. Knowl Based Syst.

[CR78] Shabani A, Abdi A, Meng L, Sylvain T (2022) Scaleformer: iterative multi-scale refining transformers for time series forecasting. arXiv:2206.04038

[CR79] Shcherbakov M, Brebels A, Shcherbakova N, Tyukov A, Janovsky T, Kamaev V (2013). A survey of forecast error measures. World Appl Sci J.

[CR80] Shen Z, Zhang Y, Lu J, Xu J, Xiao G (2020). A novel time series forecasting model with deep learning. Neurocomputing.

[CR81] Shih S-Y, Sun F-K, Lee H-Y (2019). Temporal pattern attention for multivariate time series forecasting. Mach Learn.

[CR82] Stone M (1974). Cross-validatory choice and assessment of statistical predictions. J R Stat Soc Ser B Methodol.

[CR83] Suilin A (2017) kaggle-web-traffic. Accessed: 2018-11-19. https://github.com/Arturus/kaggle-web-traffic/

[CR84] Sun F-K, Boning D S (2022) Fredo: frequency domain-based long-term time series forecasting. arXiv:2205.12301

[CR85] Svetunkov I (2021) Forecasting and analytics with adam. OpenForecast, (version: [current date]). https://openforecast.org/adam/

[CR86] Syntetos AA, Boylan JE (2005). The accuracy of intermittent demand estimates. Int J Forecast.

[CR87] Talagala T S (2020) A tool to detect potential data leaks in forecasting competitions. In: International Symposium on Forecasting 2020. https://thiyanga.netlify.app/talk/isf20-talk/

[CR88] Tashman LJ (2000). Out-of-sample tests of forecasting accuracy: an analysis and review. Int J Forecast.

[CR89] Webb GI, Hyde R, Cao H, Nguyen HL, Petitjean F (2016). Characterizing concept drift. Data Min Knowl Discov.

[CR90] Wong L (2019) Error metrics in time series forecasting. In: International Symposium on Forecasting 2019. https://isf.forecasters.org/wp-content/uploads/gravity_forms/2-dd30f7ae09136fa695c552259bdb3f99/2019/07/ISF_2019_slides.pdf

[CR91] Woo G, Liu C, Sahoo D, Kumar A, Hoi S (2022) Etsformer: exponential smoothing transformers for time-series forecasting. arXiv:2202.01381

[CR92] Wu Z, Pan S, Long G, Jiang J, Chang X, Zhang C (2020) Connecting the dots: Multivariate time series forecasting with graph neural networks. In: Proceedings of the 26th ACM SIGKDD International Conference on Knowledge Discovery & Data Mining. KDD ’20. Association for Computing Machinery, New York, NY, USA, pp 753-763

[CR93] Wu H, Xu J, Wang J, Long M (2021) Autoformer: Decomposition transformers with Auto-Correlation for long-term series forecasting. In: Advances in Neural Information Processing Systems

[CR94] Ye J, Liu Z, Du B, Sun L, Li W, Fu Y, Xiong H (2022) Learning the evolutionary and multi-scale graph structure for multivariate time series forecasting. In: Proceedings of the 28th ACM SIGKDD Conference on Knowledge Discovery and Data Mining. KDD ’22. Association for Computing Machinery, New York, NY, USA, pp 2296-2306

[CR95] Zeng A, Chen M, Zhang L, Xu Q (2022) Are transformers effective for time series forecasting?

[CR96] Zhang X, He K, Bao Y (2021). Error-feedback stochastic modeling strategy for time series forecasting with convolutional neural networks. Neurocomputing.

[CR97] Zhou Y, Zhang M, Lin K-P (2022). Time series forecasting by the novel gaussian process wavelet self-join adjacent-feedback loop reservoir model. Expert Syst Appl.

[CR98] Zhou T, Ma Z, wang X, Wen Q, Sun L, Yao T, Yin W, Jin R (2022a) Film: frequency improved legendre memory model for long-term time series forecasting. In: Advances in Neural Information Processing Systems. arXiv:2205.08897

[CR99] Zhou T, Ma Z, Wen Q, Wang X, Sun L, Jin R (2022b) FEDformer: Frequency enhanced decomposed transformer for long-term series forecasting. In: Chaudhuri K, Jegelka S, Song L, Szepesvari C, Niu G, Sabato S (eds), Proceedings of the 39th International Conference on Machine Learning. Vol. 162 of Proceedings of Machine Learning Research. PMLR, pp 27268–27286

[CR100] Zhou H, Zhang S, Peng J, Zhang S, Li J, Xiong H, Zhang W (2021) Informer: Beyond efficient transformer for long sequence time-series forecasting. In: The Thirty-Fifth AAAI Conference on Artificial Intelligence, AAAI 2021, Virtual Conference. vol 35. AAAI Press, pp 11106–11115

[CR101] Zhou T, Zhu J, Wang X, Ma Z, Wen Q, Sun L, Jin R (2022c) Treedrnet:a robust deep model for long term time series forecasting. arXiv:2206.12106

